# Sex differences in the social motivation of rats: Insights from social operant conditioning, behavioural economics, and video tracking

**DOI:** 10.1186/s13293-024-00612-4

**Published:** 2024-07-19

**Authors:** Joel S Raymond, Simone Rehn, Morgan H James, Nicholas A Everett, Michael T Bowen

**Affiliations:** 1 School of Psychology, Faculty of Science, The University of Sydney, Sydney, NSW Australia; 2 Brain and Mind Centre, The University of Sydney, 94 Mallett Street, 2050 Sydney, NSW Australia; 3 School of Life Sciences, University of Technology Sydney, Ultimo, NSW Australia; 4 Brain Health Institute, Rutgers Biomedical and Health Sciences, Rutgers University, Piscataway, NJ USA; 5 Department of Psychiatry, Robert Wood Johnson Medical School, Rutgers Biomedical Health Sciences, Rutgers University, Piscataway, NJ USA

**Keywords:** Social motivation, Social behaviour, Sex difference, Biological sex, Housing, Time-of-day, Social operant conditioning, Rat, Behavioural economics, Video tracking analysis

## Abstract

**Background:**

Social behaviour plays a key role in mental health and wellbeing, and developing greater understanding of mechanisms underlying social interaction—particularly social motivation—holds substantial transdiagnostic impact. Common rodent behavioural assays used to assess social behaviour are limited in their assessment of social motivation, whereas the social operant conditioning model can provide unique and valuable insights into social motivation. Further characterisation of common experimental parameters that may influence social motivation within the social operant model, as well as complementary methodological and analytical approaches, are warranted.

**Methods:**

This study investigated the effects of biological sex, housing condition, and time-of-day, on social motivation using the social operant model. This involved training rats to lever press (FR1) for 60-s access to a social reward (same-sex conspecific stimulus). Subjects were male and female Wistar rats, housed under individual or paired conditions, and sessions were conducted either in the mid-late light phase (ZT6-10) or early-mid dark phase (ZT13-17). A behavioural economics approach was implemented to measure social demand and the influence of stimulus partner sex (same- vs. opposite-sex stimulus) on social operant responding. Additionally, video tracking analyses were conducted to assess the degree of convergence between social appetitive and consummatory behaviours.

**Results:**

Biological sex, housing conditions, the interaction between sex and housing, and stimulus partner sex potently influenced social motivation, whereas time-of-day did not. Behavioural economics demonstrated that sex, housing, and their interaction influence both the hedonic set-point and elasticity of social demand. Video analysis of social interaction during social operant sessions revealed that social appetitive and consummatory behaviours are not necessarily convergent, and indicate potential social satiety. Lastly, oestrus phase of female experimental and stimulus rats did not impact social motivation within the model.

**Conclusions:**

Social isolation-dependent sex differences exist in social motivation for rats, as assessed by social operant conditioning. The social operant model represents an optimal preclinical assay that comprehensively evaluates social motivation and offers a platform for future investigations of neurobiological mechanisms underlying sex differences in social motivation. These findings highlight the importance of continued consideration and inclusion of sex as a biological variable in future social operant conditioning studies.

**Plain English summary:**

Humans are social creatures—our everyday interactions with others and the support this provides play a key role in our wellbeing. For those experiencing mental health conditions, people’s motivation to engage with others can wane, which can lead them to withdraw from those who support them. Therefore, to develop better treatment strategies for these conditions, we need to gain a deeper understanding of social motivation. Studying social behaviour in animals can facilitate this investigation of social motivation as it allows for a causal understanding of underlying neurobiology that is not possible in human experiments. An optimal way to study social motivation in animals is using the social operant conditioning model, where rats learn to press a lever that opens a door and allows them to interact with another rat for a short time. This study characterised the social operant model by testing whether sex, housing conditions, time-of-day, and the sex of the stimulus partner influence rats’ motivation to seek interaction with another rat. We found that female rats were more socially motivated than males, and that rats living alone were more motivated than those living with another rat; interestingly, this effect of housing affected females more than males. Regardless of sex, rats were more motivated to interact with a rat of the opposite sex. These findings provide insights into sex differences in social motivation in rats and new insights into the social operant model which will help guide future research into social motivation and other mental health conditions.

**Supplementary Information:**

The online version contains supplementary material available at 10.1186/s13293-024-00612-4.

## Highlights

Female Wistar rats exhibited greater social motivation than male rats in the social operant conditioning model.

Isolated housing produced higher social motivation than paired housing, but this isolation-induced increase was either more marked for, or only present in, female rats.

Both female and male rats demonstrate higher motivation for opposite-sex than same-sex stimulus conspecifics.

Time-of-day and oestrus phase (of female experimental and stimulus rats) did not appear to impact social motivatio.

Both between- and within-session behavioural economics in conjunction with video tracking analyses can be applied to better assess social motivation and interaction during social operant conditioning.

**Supplementary Information:**

The online version contains supplementary material available at 10.1186/s13293-024-00612-4.

## Background

Humans are fundamentally social beings [1], requiring other humans for their basic survival [2]. At an individual level, social support, bonds, and interactions play a critical role in the maintenance of positive mental health and well-being [3–6], whereas social isolation exerts a broad array of detrimental impacts on both physical and mental health [7–9]. While several mechanisms underpin the benefits of social relationships, a key component involves the social buffering of stress: social interactions that help individuals cope with and adjust to stressors that would otherwise exert greater negative impacts on their well-being [3, 10, 11]. A vital yet often neglected aspect of the relationship between social support and positive mental health is social motivation—the drive to engage in social interaction with others. Social motivation is an important consideration as, without this social drive, individuals may not engage with social support networks and would thus fail to benefit from social buffering, leaving them more vulnerable to stressors [12]. Humans and other mammals such as rats possess biobehavioural predispositions toward social interaction, including neurocircuitry encoding incentive value of social stimuli as rewarding [13–15]. Loss of social motivation and general social dysfunction are common symptoms across diverse mental disorders such as depression [16], autism spectrum disorder [13], substance use disorders [17], and schizophrenia [18]. Hence, a deeper understanding of modulatory factors underlying motivation for social interaction holds valuable transdiagnostic impact.

The use of preclinical animal models can facilitate the systematic investigation of factors influencing social behaviour and neurobiology [19–21]. While common rodent assays of social behaviour capture aspects of social interaction [22], approach [23], avoidance [24, 25], preference [26, 27], and memory [28], these are limited in their assessment of social motivation. These limitations stem from difficulties in delineating between motivation to engage socially from general locomotor activity and motivation to explore, since minimal effort is required to engage in interaction in these assays. The social operant conditioning model [29] overcomes these limitations and offers more comprehensive insight into social motivation. This model involves training rodents to perform a volitional response (lever pressing), which earns them a reward of temporary access to social interaction via a retractable door that opens revealing a conspecific (the stimulus partner rat) held within a separate compartment of the chamber [29]. Herein, rodents learn that obtaining social rewards requires performing a specific, goal-directed, previously nonsocial-related task and this allows the experimenter to directly manipulate the effort required for interaction.

The social operant model has been applied in studies of addiction and substance use [alcohol [30, 31], opioids [32–34], and psychostimulants [34–38]], feeding behaviour [32, 39, 40], pain [39], and social behaviour and neurobiology [14, 29, 31, 32, 34, 41–49] in both rats and mice. However, comparatively little research has characterised fundamental parameters influencing behaviour within the model itself [32, 43, 50]. Considering the potent influence of other commonly underappreciated biological and environmental variables in neuropsychopharmacology [51], the effects of several key parameters (and their interactions) on social motivation remain to be systematically investigated—biological sex, housing conditions, time-of-day (ToD), and the sex of the social stimuli—all of which have been shown to influence social behaviours assessed using other paradigms [52–55].

As biological sex exerts a pervasive influence on behaviour, neuroanatomy, and neurophysiology across preclinical and clinical studies [56, 57], it is imperative to investigate sex as a biological variable in preclinical research to maximise translational potential of findings [58]. Across diverse species, biological sex can modulate social motivation [59, 60], responses to social isolation [61], and the development of social neurocircuitry—e.g., the endogenous oxytocin system [62]. Previous social operant studies have included both female and male subjects [30–36, 39, 47, 48, 63], although several were not designed to detect sex differences and were consequently statistically underpowered to properly assess sex differences [32, 35, 39, 48]. Most sufficiently powered studies have not found sex differences in social operant outcomes [31, 33, 34, 36, 47, 63], however findings from a few studies suggest this absence of sex differences is not definitive and may depend on outcomes assessed [30], interactions with other factors [32, 64], and species tested [43].

Social housing context is another key experimental parameter requiring attention in the social operant model [15, 65, 66], particularly in conjunction with biological sex. At present, studies that have explored the impact of social housing on social operant responding indicate an isolation-induced enhancement of social motivation [31, 32, 43, 48, 49]. However, these studies have been conducted in solely female [32, 43, 49] or male [31] subjects, or the small sample size did not allow for statistical investigation of a sex difference in this housing effect [48]. As such, there is currently little to no indication whether this social isolation-induced increase in social motivation generalises across both sexes and there is a need to investigate the potential for a *Sex* x *Housing* interaction.

In laboratory settings, rodents are typically housed under 12-h light/dark cycles [51], demonstrating crepuscularity (i.e., most activity at transitions of the light cycle) and circadian patterns of more activity during the dark phase [67–69] and more sleep during the light phase [70, 71]. ToD can influence motivated behaviour in self-administration models [72–74] and natural circadian variations in spontaneous social behaviour have been previously identified [75]; this influence of ToD may extend to motivation for social reward. However, since rodents can adjust their behavioural patterns based on social entrainment cues [76], it is possible that rodent social behavioural assays are insensitive to effects of ToD [54]. Previous social operant studies have been conducted during both dark and light phases; however, several studies report no information about ToD and no studies have systematically investigated the impact of ToD on social motivation or potential *Sex* x *ToD* interactions using social operant conditioning.

Social interactions between rodent conspecifics are highly species- and sex-dependent [77–79]; hence it is necessary to investigate how sex of stimulus partners influences social motivation for both sexes within the social operant model. Male rats typically display a preference for female (i.e., opposite-sex) over male (i.e., same-sex) conspecifics [55], and this opposite-sex preference was also identified in juvenile female rats during novel play interactions [80]. Only one study has examined stimulus partner sex in the social operant model, demonstrating that male but not female rats exhibited preference for opposite-sex over same-sex stimulus partners [64]. One other study in both male and female mice used juvenile males as stimuli, but no sex difference information was reported [47], and the remaining previous social operant studies have all used same-sex stimulus conspecifics. Hence, further investigation of the influence of stimulus sex on both male and female subjects in social operant conditioning is warranted.

While previous studies have employed behavioural economics approaches to comprehensively assess motivation for drug and food rewards [81–84], few studies have used behavioural economics to investigate motivation for social reward [37, 40, 64]. Behavioural economics constitutes a translationally-relevant approach to assessing motivation that offers advantages over using fixed-ratio and progressive-ratio responding as indicators of motivation: facilitating an evaluation of both hedonic motivation for rewards and how motivation changes with increases in reward cost [81]. All previous social operant studies using behavioural economics have used between-session procedures, which have limited capacities to investigate pharmacotherapies and neural mechanisms due to the long duration of testing required [85]. These limitations can be addressed using a within-session procedure [85], however—to date—no social operant studies have implemented this. Given that the influence of biological sex and other experimental parameters on social motivation may depend on the effort required to obtain social rewards, behavioural economics represents an optimal method to implement alongside social operant conditioning.

Unlike drug self-administration models involving intravenous delivery of reward [86], social operant conditioning involves a unique consummatory aspect wherein subjects’ engagement with the reward (i.e., social interaction) is highly complex. Since the reinforcer in this model constitutes temporary access to a social stimulus, subjects can receive a social reward (i.e., social door opens) and spend anywhere between 0 and 100% of this open-door period engaging in interaction with the stimulus partner (i.e., consuming the reward). This represents a critical distinction between appetitive (i.e., motivation for social interaction) and consummatory (i.e., engaging in social interaction itself) processes [87] within the social operant model that cannot be assessed by purely lever press-related outcomes. As experimental parameters (e.g., biological sex) may influence social appetitive and consummatory processes in convergent or divergent ways, the complementary assessment of social behaviour using video recording in conjunction with operant measures of motivation is warranted.

The current study employed the social operant conditioning model, behavioural economics approaches, and video tracking analyses to investigate (1) how biological sex, housing conditions, ToD, stimulus partner sex, and corresponding interactions between these parameters and biological sex impact social motivation; (2) whether these effects depend on effort required to obtain social rewards; and (3) whether these parameters impact social appetitive and consummatory behaviours differently. Based on the aforementioned literature, it was hypothesised that (a) female rats would demonstrate greater social motivation than males; (b) isolated rats would show higher social motivation than pair-housed rats; (c) rats would show greater social motivation during the dark compared to light phase; and (d) male but not female rats would show greater motivation for opposite-sex social stimuli.

## Methods

### Subjects

Female and male (*N* = 64; *n* = 32/sex) young adult (6-week-old) Wistar rats, weighing on average 205 g (F) and 293 g (M) on arrival, were sourced from Animal Resources Centre (Perth, WA, Australia). Rats were initially housed in pairs; half of the experimental rats were later transitioned to individual housing, as detailed below. Stimulus rats (*n* = 8/sex) were always maintained in paired housing. Rats were housed in standard IVC cages in a temperature- and humidity- controlled colony room (21.3 ± 0.1 °C; 61.7 ± 0.5%) under standard light cycle conditions (12 L:12D; lights on at 0600, ZT0) and were given *ad libitum* access to standard laboratory chow and water. Social operant testing was conducted either from mid-late light phase (1–4 pm; ZT6-10) or from early-mid dark phase (6–8 pm; ZT12-14) unless otherwise indicated.

### Apparatus

All social operant conditioning procedures were conducted using social self-administration chambers for rats manufactured by Med-Associates Inc. [29]. Briefly, these chambers were equipped with a houselight situated above two extendable levers with a distinct cue light located above each lever. These were positioned opposite from an aluminium grill that covered a retractable guillotine door between the main experimental chamber and a smaller stimulus chamber. Social operant sessions were operated using custom Med-PC code and software (Med-Associates Inc., version V). Operant chambers were located inside sound- and light-attenuating boxes. Videos of all social operant sessions were recorded using ceiling-mounted infrared security cameras (Swann, SWDVK-845,808V), and videos were recorded at 15 frames per second (928 wide x 576 high resolution, 2098 bps).

### Design

All testing was conducted over consecutive sessions using mixed, repeated-measures, counterbalanced designs. Independent variables included *biological sex* (male vs. female), *housing condition* (paired vs. isolated), *ToD* (mid-late light vs. early-mid dark phase) as between-subjects variables, and *stimulus sex* (same vs. opposite) and *experimental session* as within-subject variables. All rats were randomly allocated to experimental or stimulus roles, as well as to housing and ToD conditions using a random number generator approach (Microsoft Excel, RAND function). While the sex of experimental rats was kept consistent within individual operant chambers, active/inactive levers and cue light colours were counterbalanced across all conditions. During all experimental phases, stimulus rats were constantly cycled between chambers (i.e., experimental rats interacted with the same stimulus rat every eighth session) to maintain some novelty of the social stimulus each session.

#### Phase 1– Acquisition of social operant conditioning (1a) and ToD switch (1b)

**Fig. 1:**
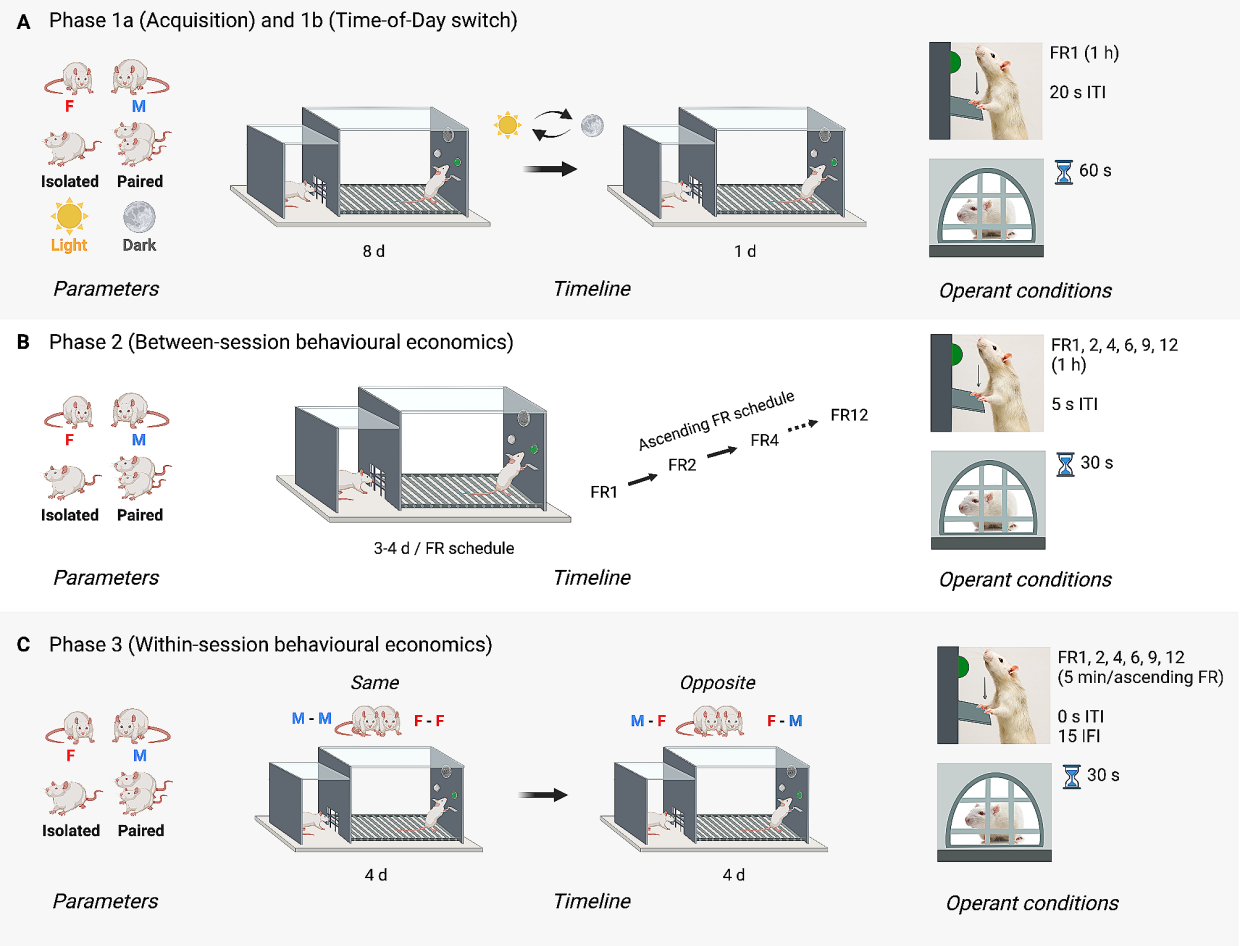
Experimental design and procedures. Schematic of the experimental design, timeline, and procedures involved in (**A**) Phase 1a– Acquisition and 1b– Time-of-Day switch; (**B**) Phase 2– Between-session behavioural economics; and (**C**) Phase 3– Within-session behavioural economics. During Phase 1a, the impact of experimental parameters [biological sex (female vs. male), housing condition (isolated vs. paired), and ToD (light vs. dark phase)] on acquisition of social operant responding were assessed over the course of eight days. This was followed by an additional social operant session involving the switching of ToD under which rats were tested (Phase 1b). Operant conditions for Phase 1a involved 1-h daily sessions of FR1 schedule, each social reward involved temporary access to a same-sex conspecific for 60 s via a retractable door, and each reward delivery was followed by a 20-s intertrial interval. For Phase 2, the influence of biological sex, housing condition, and effort required for social reward on social motivation was investigated using a between-session behaviour economics approach. Operant conditions for Phase 2 involved 3–4 × 1-h daily social operant sessions at each FR schedule (FR1, 2, 4, 6, 9, 12), social reward was reduced to 30-s access to social stimuli, and 5-s intertrial intervals following delivery of social reward. During Phase 3, the effect of parameters [biological sex, housing condition, stimulus sex (same vs. opposite)] on social motivation was examined using a within-session behavioural economic paradigm. This involved four days of testing with same-sex stimuli followed by four days of opposite-sex stimuli. Each session for Phase 3 involved successive 5-min bins of time for each FR schedule (FR1, 2, 4, 6, 9, 12) presented in an ascending order and separated by 15-s intervals (interFR intervals); social reward was 30-s access to social stimuli, and 0-s intertrial intervals following the delivery of a social reward. Note that for Phase 2 and 3, ToD was variable was removed—all social operant testing was conducted during the early-mid dark phase—and only the top-half of highest responders were included. d– Day(s); F– female; FR– fixed ratio; IFI– interFR-interval; ITI– intertrial interval; M– male, opposite– opposite-sex stimulus, same– same-sex stimulus. This figure was created with BioRender.com.

Phase 1a characterised the influence of sex, housing condition, and ToD on the acquisition of social operant responding (Fig. 1A). A *Sex* x *Housing* x *ToD* x *session* design was employed (*n* = 8 per factorial condition) and testing was conducted intermittently; two cohorts alternated testing every second day, and each cohort involved four 1-hour sessions across the day. During Phase 1b, ToD conditions were swapped for all rats, but the same factorial design was maintained.

#### Phase 2– Between-session social operant behavioural economics

Phase 2 explored the influence of sex, housing condition, and effort (fixed ratio (FR) schedule) on social motivation using a continuous (daily) between-session behavioural economics approach (Fig. 1B). Because ToD did not appear to impact social operant responding in Phase 1, all testing in Phase 2 was conducted during the early-mid dark phase (ZT12-18). Additionally, only half of the rats from Phase 1 (*n* = 32) were included from Phase 2 onwards; inclusion of the top 50% of responders was determined using the highest average number of social rewards obtained across the sessions of Phase 1, and selecting the top 50% (*n* = 4) from each *Sex* x *Housing* x *ToD* condition and operant chamber (i.e., to maintain identical chamber conditions). Rats from each *Sex* x *Housing* condition were counterbalanced across order of session, and mean rewards did not differ greatly between each of the four daily runs (Range = 18.5–24.5). For further details, about inclusion and exclusion of subjects from Phases 2 and 3, see supplemental material Sect. 1, Figures S1-S3, and Table S1.

#### Phase 3– Within-session social operant behavioural economics

Phase 3 investigated the influence of sex, housing condition, and stimulus sex on social motivation using a novel within-session behavioural economics approach (Fig. 1C). Sessions were conducted intermittently (every other day) to enhance social motivation via reducing session-to-session social satiety, and four sessions of each *stimulus sex* condition were conducted with same-sex sessions preceding opposite-sex sessions.

### Procedure

#### Habituation

Upon arrival, rats were allowed one week of acclimatisation to the facility prior to any experimental procedures. Subsequently, rats were rehoused as isolated or paired—according to randomisation—for one week prior to the commencement of social operant conditioning. During this week, all rats were handled for 3 min each on two different days to habituate rats to handling and prevent novelty- and handling-induced stress during experimental operant procedures. One day prior to starting social operant conditioning, both cohorts of experimental rats were habituated to operant chambers for 15-min sessions from ZT6.5-12.5. During these sessions, rats were habituated to the experimental compartment of operant chambers: no levers were extended, only the houselight was illuminated, the exhaust fan was active, and no social stimulus was present in the stimulus compartment. Additionally, 15-min habituation sessions for stimulus rats were conducted by placing them in the stimulus compartment in the absence of an experimental rat in the experimental compartment.

#### Phase 1– Acquisition and ToD switch

Acquisition (Phase 1a) involved eight intermittent social operant conditioning sessions lasting 70 min in duration, where rats were trained on a fixed ratio (FR) 1 reinforcement schedule (Fig. 1A). Ten minutes after rats were placed in the operant chamber, the houselight was illuminated (remaining illuminated for the remainder of the session), and both active and inactive levers were extended into the experimental compartment. Completion of the FR1 reinforcement requirement (i.e., one lever press of the active lever) led to illumination of the cue-light above the active lever, activation of a pure tone (2900 Hz, 78 dB, 5 s duration), and opening of the social door for 60 s. After 5 s, the active lever was retracted, however the inactive lever was always available. After the assigned 60 s elapsed, the social door closed, and a 20 s inter-trial interval (ITI) began. This process repeated until the end of the session was reached (60 min). For Phase 1b, operant sessions were conducted in an identical manner to Phase 1a except that the ToD that rats were tested was switched from the original 8 sessions (i.e., from mid-late light phase to early-mid dark phase and vice versa). Operant chambers were cleaned with F10SC between each run of each session.

#### Phase 2– Between-session behavioural economics

Across daily sessions, effort required to earn a social reward was increased according to the following pattern: 3 x FR1 sessions, 3 x FR2 sessions, 4 x FR4 sessions, 3 x FR6 sessions, 3 x FR9 sessions, and 3 x FR12 sessions (Fig. 1B). Procedures were identical to Phase 1, except for the following modifications: upon reward delivery, both levers were immediately retracted, and the social door was opened for 30 s instead of 60 s. After this time elapsed, the social door closed, and a 5-s ITI was employed. This process repeated until the end of the session was reached.

#### Phase 3– Within-session behavioural economics

Within-session procedures involved intermittent sessions (every other day) where the effort required to complete the FR reinforcement requirement was escalated within the same session following an ascending pattern: FR1, FR2, FR4, FR6, FR9, and FR12 (Fig. 1C). In these sessions, following the 10-min starting period, the houselight was illuminated and both levers were extended for a 5 min period of a FR1 reinforcement schedule. Completion of the FR requirement led to the social door opening for 30 s after which, the door closed, and the levers were immediately re-extended. After 5 min, a 15 s timeout period was employed involving a closed social door, no houselight and both levers retracted, after which another 5 min period of the successive FR schedule began. If a social reward was obtained within less than 30 s of the 5 min FR session ending, then a full 30 s reward was allowed, followed by a delayed 15 s timeout. Four sessions were conducted with same-sex stimuli followed by four sessions with opposite-sex stimuli.

### Oestrus phase determination

During Phase 3, oestrus phase was determined for female rats immediately after each social operant session. Briefly, rats were restrained and the tip of a plastic Pasteur pipette containing approximately 0.15 mL of saline (0.9% w/v) was carefully inserted into the vagina (≤ 1 cm insertion depth). The saline was expelled to gently flush the vagina, and then vaginal fluid was collected back into the pipette. Vaginal fluid (2–3 drops) from each rat was placed onto individually labelled glass slides and then unstained samples were examined under a light microscope using a 10 x objective lens. Additionally, photographs (2–5 images) of each sample were taken using a microscope digital camera (M500 BASE, Levenhuk) and software (ToupView, Levenhuk). Oestrus phase was determined based on the relative proportion of cell types (i.e., epithelial, cornified and leukocytes) present in the sample, as previously described [88].

### Data processing

Outcomes of interest extracted from Med-PC (Med Associates Inc.) were social rewards obtained, alongside active and inactive lever presses. Outcomes extracted from video tracking analyses with DeepLabCut were locomotion, total time rats spent with their nose in open and closed doors, and the proportion of open- and closed-door time that rats spent with their noses in the door.

#### Behavioural economics analysis

For between-session behavioural economic analysis (Phase 2), an adjusted mean value of social rewards obtained (i.e., active lever presses divided by FR schedule required) was calculated for each rat across the sessions of each FR schedule (i.e., price point). For within-session behavioural economic analysis (Phase 3), the number of social rewards was calculated for each FR price point within each session and then averaged across the four sessions of each stimulus sex condition. Subsequently, exponential demand curves [89] were fit to each rat’s data and two outcomes of interest were extracted: demand elasticity (α) and demand at null cost (Q_0_). Briefly, α represents the rate of reduction in social reward seeking as the cost of social interaction increases (demand elasticity); lower α values indicate that responding for social reward is less sensitive to increases in cost, implying higher social motivation. Q_0_ reflects preferred level of social reward at a theoretical null cost; this may be interpreted as the hedonic set-point for social reward and can represent a complementary but independent indicator of motivation from α [89].

#### Video recording, pose estimation, and kinematic analyses

For tracking of individual body and chamber parts, we trained a DeepLabCut (version 2.3) model using a ResNet-50 based neural network with default parameters on 1384 frames taken from 155 videos of ∼ 40 animals for 800,000 iterations. The training data encompassed a diverse range of behavioural phenotypes, experimental parameters, and lighting conditions to ensure robust translation of the network. Additionally, sparse but important periods in videos such as cue light illumination were isolated using custom video-editing scripts [90], and labelled frames from these periods were over-represented in the final model. Evaluation of model performance revealed that the test error was 6.6 pixels, relative to an error of 3.2 pixels for the training data, indicating that suitable average tracking performance was achieved given the 928 × 576 resolution [91]. This network was then used to analyse novel videos from similar experimental settings.

Body and chamber part coordinates predicted by DeepLabCut were analysed using custom Python scripts in Jupyter Notebook [92, 93]. These scripts are available for other researchers on a collaborative basis. In brief, the position and Euclidean distance of all body parts (nose, left ear, right ear, neck-base, body-centre, tail-base, tail-mid, tail-tip) were calculated for each frame in relation to each chamber part (four corners of floor, food cup, four corners of social door, centre of each extended lever, centre of each cue light). As the ceiling of the operant chamber would sometimes occlude body part tracking when the animal was centred in the arena for brief periods, we filtered all tracking points to only include those which exceeded 0.6 tracking likelihood, and linearly interpolated the missing tracking points (< 0.6 likelihood). These computed values were then used to operationalise behavioural events, such as interaction between the nose and: social door, levers, cue lights, and food cup.

Additionally, as the door’s status (open/closed) could not be directly tracked with DeepLabCut, the door status was annotated based on the illumination of cue lights (using a tracking likelihood cut-off of > 0.9 for > 1 s) which signalled the beginning of the 30- or 60-s open door period. To further automate analysis of these ∼1800 videos, the ‘start time’ of the operant session within each video was annotated by identification of the first 1-s span where both levers had high (> 0.9) tracking likelihood scores, indicating that they had been extended into the chamber at the initiation of the Med Associates program. For analyses of Phase 3 (within-session behavioural economics), we incorporated timestamps from Med Associates output files, to align the video kinematic analysis with each fixed-ratio bin.

#### Oestrus phase

For data analysis, oestrus phases were dichotomised into proestrus/oestrus (P/O) or metoestrus/dioestrus (M/D) categories. This was based on proestrus and oestrus phases representing a period of elevated sex hormone levels (e.g., oestrogen and progesterone) compared to metoestrus and dioestrus, which represented a period of relatively lower hormone levels [94, 95]. Social operant outcomes were averaged across the four sessions of an oestrus category to form a mean for P/O and M/D, and these were used for statistical analysis. Since rats exhibited a large range of baseline social motivation, only paired data (i.e., rats experiencing both P/O and M/D conditions) were included in within-subject statistical analyses.

### Statistical analysis

Statistical analyses were conducted using IBM SPSS software (version 25) and data visualisation was conducted using GraphPad Prism (version 9.5.1). The level of statistical significance for all tests was *p* <.05. Greenhouse-Geisser corrections were applied whenever violations of sphericity were detected. Bonferroni corrections were applied to all planned simple effects contrasts of *Sex* x *Housing*. For behavioural economics analyses, rats with demand curves that fit the observed data with an R^2^ value < 0.6 were excluded (see figure legends for details on attrition). Latency to first active lever press and demand elasticity data values were log transformed to address issues with normality and heteroscedasticity [96]. Across all experimental phases, data attrition occurred for outcomes dependent on video recording due to either poor quality/lost video files or due to rats not achieving at least one reward (i.e., proportion of open door time cannot be computed if zero).

The statistical tests conducted for each experimental phase and outcome are summarised below in Table 1. During Phase 1a, polynomial trend analysis was conducted for *Session* and *Time* to identify if linear and quadratic trends were present across acquisition sessions and within sessions, respectively. For Phase 1b, within-subject comparison was conducted between the final acquisition session 8 (pre-switch) to session 9 (post-switch). For Phase 2, polynomial trend analysis was conducted for *FR schedule* to identify if linear and quadratic trends were present across increasing effort requirements. Further, to assess the strength and nature of the relationship between between-session demand at null cost and between-session demand elasticity, a Pearson’s correlation coefficient was computed for the entire cohort, as well as split by *Sex*. For Phase 3, Pearson’s correlation coefficients were computed to assess the strength and nature of the relationships between (i) within-session demand at null cost and within-session demand elasticity, (ii) between-session demand at null cost and within-session demand at null cost, and (iii) between-session demand elasticity and within-session demand elasticity.

**Table 1:** Summary of statistical analyses

Phase	Statistical test(s)	Outcome variables
**1a** Acquisition	Repeated measures ANOVAs Four-way: 2 × 2 × 2 × (8) *Sex* x *Housing* x *ToD* x (*Session*) • Polynomial trend analysis on *Session* • Planned simple effects contrasts on *Sex* x *Housing*	• Social rewards • Difference in active-inactive lever presses • Latency to first active lever press • Locomotion • Total time with nose in open and closed door • Proportion of open- and closed-door time with nose in door
	Repeated measures ANOVAs Four-way: 2 × 2 × 2 × (8) *Sex* x *Housing* x *ToD* x (*Time*) • Polynomial trend analysis on *Time* • Planned simple effects contrasts on *Sex* x *Housing*	• Within-session time course mean rewards • Within-session proportion of open door time with nose in door
**1b** ToD switch	Repeated measures ANOVAs Four-way: 2 × 2 × 2 × (2) *Sex* x *Housing* x *ToD* x (*Session*)	• Social rewards • Difference in active-inactive lever presses • Latency to first active lever press • Locomotion • Total time with nose in open and closed door • Proportion of open- and closed-door time with nose in door
**2** Between-session behavioural economics	Repeated measures ANOVAs Three-way: 2 × 2 × (6) *Sex* x *Housing* x (*FR schedule*) • Polynomial trend analysis on *FR schedule*	• Mean adjusted social rewards • Total time with nose in open and closed door • Proportion of open- and closed-door time with nose in door
	Univariate ANOVAs Two-way: 2 × 2 *Sex* x *Housing*	• Social demand at null cost • Social demand elasticity
**3** Within-session behavioural economics	Repeated measures ANOVAs Three-way: 2 × 2 × (2) *Sex* x *Housing* x (*Stimulus sex*) Two-way: 2 × (2) *Stimulus oestrus phase* x (*Sex*) Two-way: (2 × 2) (*Experimental oestrus phase* x *Stimulus sex*)	• Mean social rewards • Social demand at null cost • Social demand elasticity • Total time with nose in open and closed door • Proportion of open- and closed-door time with nose in door

## Results

### Acquisition (Phase 1a)

**Fig. 2:**
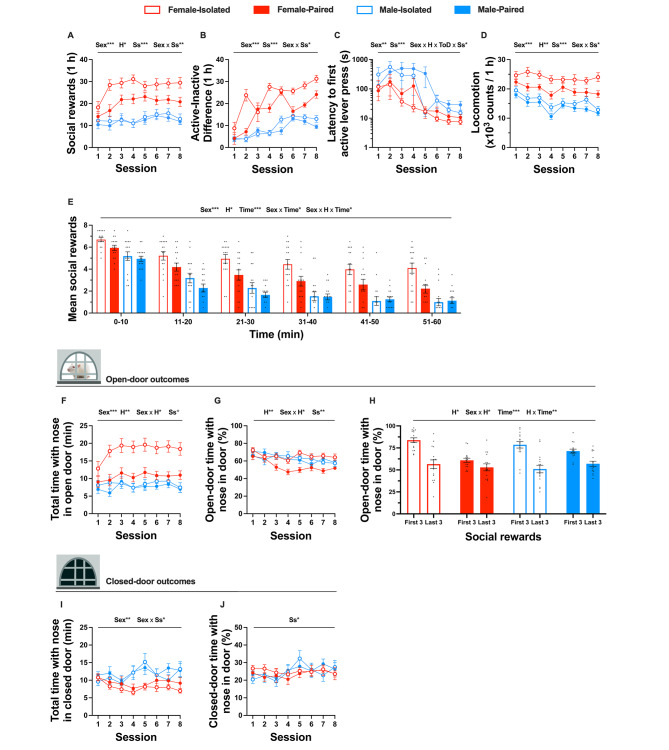
The influence of sex and housing conditions on outcomes of acquisition in social operant conditioning. Effects of experimental parameters of biological sex and housing conditions on social rewards (**A**), difference in active-inactive lever presses (**B**), latency to first active lever press (**C**), locomotor activity (**D**), within-session time course of mean social rewards averaged over sessions 7 and 8 (**E**), total time with nose in open door (**F**), proportion of open-door time with nose in door (**G**), within-session proportion of open-door time with nose in door averaged over sessions 7 and 8 (**H**), total time with nose in closed door (**I**), and proportion of closed-door time with nose in door (**J**). Sample sizes were *n* = 8 per factorial condition, except for locomotion, total time with nose in open door, total time with nose in closed door, and proportion of closed-door time with nose in door (F-Iso-L: *n* = 7, F-Pair-L: *n* = 7, F-Iso-D: *n* = 6, F-Pair-D: *n* = 6, M-Iso-L: *n* = 7, M-Pair-L: *n* = 8, M-Iso-D: *n* = 6, M-Pair-D: *n* = 6); and proportion of open-door time with nose in door (F-Iso-L: *n* = 7, F-Pair-L: *n* = 7, F-Iso-D: *n* = 6, F-Pair-D: *n* = 6, M-Iso-L: *n* = 6, M-Pair-L: *n* = 8, M-Iso-D: *n* = 6, M-Pair-D: *n* = 4). Data represent mean values ± S.E.M. and individual data points represent individual subject data. Statistical significance is indicated by the following: Sex– biological sex; H– housing condition; ToD– time-of-day; Ss– session, Time– timepoint during session. Level of statistical significance is indicated by the number of * symbols: one– *p* <.05, two– *p* <.01, three– *p* <.001

In Phase 1a, we sought to determine the effects of biological sex, housing conditions, and ToD on social motivation and related outcomes during acquisition of social operant conditioning under a FR1 schedule of reinforcement. Table 2 and Fig. 2 summarise the statistical analyses and results from Phase 1a; note that, as no significant main effect of ToD or interactions with ToD were found for social rewards, results relating to ToD are illustrated in supplemental Figure S4.

**Table 2:** Summary of results from Phase 1a (Acquisition) statistical analyses

Outcome	Effect	F statistic	p-value	Partial η^2^
*Acquisition (Phase 1a)*				
Social rewards	Sex	*F*_(1, 56)_ = 43.01	< 0.001*	0.43
	Housing	*F*_(1, 56)_ = 6.10	0.017*	0.10
	ToD	*F*_(1, 56)_ = 0.34	0.561	0.01
	Session	F_(4.085, 228.779)_ = 8.70	< 0.001*	0.13
	Sex x Housing	*F*_(1, 56)_ = 3.53	0.066	0.06
	Housing: Females^a^	*F*_(1, 56)_ = 9.45	0.003^#^	0.14
	Housing: Males^a^	*F*_(1, 56)_ = 0.18	0.677	< 0.01
	Sex x ToD	*F*_(1, 56)_ < 0.01	0.977	< 0.01
	Sex x Session	F_(4.085, 228.779)_ = 4.71	0.001*	0.08
	Housing x ToD	*F*_(1, 56)_ = 0.24	0.625	< 0.01
	Housing x Session	F_(4.085, 228.779)_ = 0.78	0.539	0.01
	ToD x Session	F_(4.085, 228.779)_ = 1.96	0.101	0.03
	Sex x Housing x ToD	*F*_(1, 56)_ = 0.11	0.743	< 0.01
	Sex x Housing x Session	F_(4.085, 228.779)_ = 0.83	0.513	0.02
	Sex x ToD x Session	F_(4.085, 228.779)_ = 0.84	0.501	0.02
	Housing x ToD x Session	F_(4.085, 228.779)_ = 0.30	0.879	0.01
	Sex x Housing x ToD x Session	F_(4.085, 228.779)_ = 1.41	0.230	0.03
Difference in active-inactive lever presses	Sex	*F*_(1, 56)_ = 22.01	< 0.001*	0.28
	Housing	*F*_(1, 56)_ = 2.92	0.093	0.05
	ToD	*F*_(1, 56)_ = 0.46	0.501	0.01
	Session	F_(3.748, 209.867)_ = 13.60	< 0.001*	0.20
	Sex x Housing	*F*_(1, 56)_ = 1.38	0.244	0.02
	Housing: Females^a^	*F*_(1, 56)_ = 4.16	0.046	0.07
	Housing: Males^a^	*F*_(1, 56)_ = 0.14	0.708	< 0.01
	Sex x ToD	*F*_(1, 56)_ = 0.04	0.840	< 0.01
	Sex x Session	F_(3.748, 209.867)_ = 3.14	0.018*	0.05
	Housing x ToD	*F*_(1, 56)_ = 1.26	0.267	0.02
	Housing x Session	F_(3.748, 209.867)_ = 1.13	0.344	0.02
	ToD x Session	F_(3.748, 209.867)_ = 2.18	0.077	0.04
	Sex x Housing x ToD	*F*_(1, 56)_ = 0.54	0.817	< 0.01
	Sex x Housing x Session	F_(4.332, 242.604)_ = 1.38	0.245	0.02
	Sex x ToD x Session	F_(4.332, 242.604)_ = 0.35	0.830	0.01
	Housing x ToD x Session	F_(4.332, 242.604)_ = 0.37	0.816	0.01
	Sex x Housing x ToD x Session	F_(4.332, 242.604)_ = 1.54	0.195	0.03
Latency to active lever first press *(log transform)*	Sex	*F*_(1, 56)_ = 10.52	0.002*	0.16
	Housing	*F*_(1, 56)_ = 1.74	0.192	0.03
	ToD	*F*_(1, 56)_ = 2.75	0.103	0.05
	Session	F_(4.904, 274.617)_ = 17.70	< 0.001*	0.24
	Sex x Housing	*F*_(1, 56)_ = 0.2	0.902	< 0.01
	Housing: Females^a^	*F*_(1, 56)_ = 0.72	0.401	0.01
	Housing: Males^a^	*F*_(1, 56)_ = 1.04	0.312	0.02
	Sex x ToD	*F*_(1, 56)_ = 1.60	0.212	0.03
	Sex x Session	F_(4.904, 274.617)_ = 0.15	0.980	< 0.01
	Housing x ToD	*F*_(1, 56)_ = 0.12	0.283	0.02
	Housing x Session	F_(4.904, 274.617)_ = 1.41	0.223	0.03
	ToD x Session	F_(4.904, 274.617)_ = 1.64	0.150	0.03
	Sex x Housing x ToD	*F*_(1, 56)_ = 2.78	0.074	0.06
	Sex x Housing x Session	F_(4.904, 274.617)_ = 0.86	0.505	0.02
	Sex x ToD x Session	F_(4.904, 274.617)_ = 0.51	0.767	0.01
	Housing x ToD x Session	F_(4.904, 274.617)_ = 1.15	0.333	0.02
	Sex x Housing x ToD x Session	F_(4.904, 274.617)_ = 2.31	0.046*	0.04
Locomotion	Sex	*F*_(1, 45)_ = 40.37	< 0.001*	0.47
	Housing	*F*_(1, 45)_ = 10.70	0.002*	0.19
	ToD	*F*_(1, 45)_ = 1.56	0.218	0.03
	Session	F_(5.342, 240.372)_ = 9.94	< 0.001*	0.18
	Sex x Housing	*F*_(1, 45)_ = 0.52	0.653	< 0.01
	Housing: Females^a^	*F*_(1, 45)_ = 7.85	0.007^#^	0.15
	Housing: Males^a^	*F*_(1, 45)_ = 0.37	0.076	0.07
	Sex x ToD	*F*_(1, 45)_ = 0.22	0.882	< 0.01
	Sex x Session	F_(5.342, 240.372)_ = 2.36	0.037*	0.05
	Housing x ToD	*F*_(1, 45)_ = 0.02	0.899	< 0.01
	Housing x Session	F_(5.342, 240.372)_ = 1.79	0.110	0.04
	ToD x Session	F_(5.342, 240.372)_ = 2.92	0.012*	0.06
	Sex x Housing x ToD	*F*_(1, 45)_ = 0.51	0.778	0.01
	Sex x Housing x Session	F_(5.342, 240.372)_ = 1.70	0.198	0.04
	Sex x ToD x Session	F_(5.342, 240.372)_ = 0.58	0.725	0.01
	Housing x ToD x Session	F_(5.342, 240.372)_ = 0.39	0.866	0.01
	Sex x Housing x ToD x Session	F_(5.342, 240.372)_ = 0.84	0.532	0.02
*Within-session time course*: Mean social rewards	Sex	*F*_(1, 56)_ = 37.30	< 0.001*	0.40
	Housing	*F*_(1, 56)_ = 6.14	0.016*	0.10
	ToD	*F*_(1, 56)_ = 0.66	0.419	0.01
	Time	F_(3.955, 221.465)_ = 144.94	< 0.001*	0.72
	Sex x Housing	*F*_(1, 56)_ = 2.79	0.100	0.05
	Housing: Females^a^	*F*_(1, 56)_ = 8.60	0.005^#^	0.13
	Housing: Males^a^	*F*_(1, 56)_ = 0.33	0.571	0.01
	Sex x ToD	*F*_(1, 56)_ = 0.06	0.803	< 0.01
	Sex x Time	F_(3.955, 221.465)_ = 2.68	0.033*	0.46
	Housing x ToD	*F*_(1, 56)_ = 0.49	0.486	0.01
	Housing x Time	F_(3.955, 221.465)_ = 0.89	0.468	0.02
	ToD x Time	F_(3.955, 221.465)_ = 0.92	0.454	0.02
	Sex x Housing x ToD	*F*_(1, 56)_ = 0.95	0.333	0.02
	Sex x Housing x Time	F_(3.955, 221.465)_ = 2.53	0.042*	0.04
	Sex x ToD x Time	F_(3.955, 221.465)_ = 1.51	0.201	0.03
	Housing x ToD x Time	F_(3.955, 221.465)_ = 0.49	0.741	0.01
	Sex x Housing x ToD x Time	F_(3.955, 221.465)_ = 0.78	0.536	0.01
Total time with nose in open door	Sex	*F*_(1, 45)_ = 24.82	< 0.001*	0.36
	Housing	*F*_(1, 45)_ = 11.83	0.001*	0.21
	ToD	*F*_(1, 45)_ = 0.78	0.381	0.02
	Session	*F*_(4.298, 193.402)_ = 2.68	0.030*	0.06
	Sex x Housing	*F*_(1, 45)_ = 5.07	0.029*	0.10
	Housing: Females^a^	*F*_(1, 45)_ = 15.96	< 0.001^#^	0.26
	Housing: Males^a^	*F*_(1, 45)_ = 0.72	0.402	0.02
	Sex x ToD	*F*_(1, 45)_ = 0.03	0.860	< 0.01
	Sex x Session	*F*_(4.298, 193.402)_ = 1.90	0.107	0.04
	Housing x ToD	*F*_(1, 45)_ = 0.03	0.875	< 0.01
	Housing x Session	*F*_(4.298, 193.402)_ = 1.44	0.219	0.03
	ToD x Session	*F*_(4.298, 193.402)_ = 1.55	0.185	0.03
	Sex x Housing x ToD	*F*_(1, 45)_ = 0.02	0.890	< 0.01
	Sex x Housing x Session	*F*_(4.298, 193.402)_ = 0.45	0.790	0.01
	Sex x ToD x Session	*F*_(4.298, 193.402)_ = 1.34	0.255	0.03
	Housing x ToD x Session	*F*_(4.298, 193.402)_ = 0.24	0.926	0.01
	Sex x Housing x ToD x Session	*F*_(4.298, 193.402)_ = 0.32	0.874	0.01
Proportion of open-door time with nose in door	Sex	*F*_(1, 42)_ = 0.03	0.616	0.01
	Housing	*F*_(1, 42)_ = 8.00	0.007*	0.16
	ToD	*F*_(1, 42)_ = 5.18	0.028*	0.11
	Session	F_(5.269, 221.309)_ = 4.01	0.001*	0.09
	Sex x Housing	*F*_(1, 42)_ = 6.21	0.017*	0.13
	Housing: Females^a^	*F*_(1, 42)_ = 15.17	< 0.001^#^	0.27
	Housing: Males^a^	*F*_(1, 42)_ = 0.05	0.820	< 0.01
	Sex x ToD	*F*_(1, 42)_ = 0.02	0.904	< 0.01
	Sex x Session	F_(5.269, 221.309)_ = 1.38	0.230	0.03
	Housing x ToD	*F*_(1, 42)_ = 0.06	0.813	< 0.01
	Housing x Session	F_(5.269, 221.309)_ = 1.40	0.225	0.03
	ToD x Session	F_(5.269, 221.309)_ = 1.28	0.270	0.03
	Sex x Housing x ToD	*F*_(1, 42)_ = 1.84	0.182	0.04
	Sex x Housing x Session	F_(5.269, 221.309)_ = 0.28	0.929	0.01
	Sex x ToD x Session	F_(5.269, 221.309)_ = 0.59	0.713	0.01
	Housing x ToD x Session	F_(5.269, 221.309)_ = 0.23	0.954	0.01
	Sex x Housing x ToD x Session	F_(5.269, 221.309)_ = 1.21	0.307	0.03
*Within-session* proportion of open-door time with nose in door	Sex	*F*_(1, 56)_ = 0.14	0.713	< 0.01
	Housing	*F*_(1, 56)_ = 7.01	0.011*	0.11
	ToD	*F*_(1, 56)_ = 2.09	0.154	0.04
	Time	F_(1, 56)_ = 74.41	< 0.001*	0.57
	Sex x Housing	*F*_(1, 56)_ = 5.92	0.018*	0.10
	Housing: Females^a^	*F*_(1, 56)_ = 12.91	0.001^#^	0.19
	Housing: Males^a^	*F*_(1, 56)_ = 0.02	0.880	< 0.01
	Sex x ToD	*F*_(1, 56)_ = 0.16	0.687	< 0.01
	Sex x Time	F_(1, 56)_ = 0.59	0.445	0.01
	Housing x ToD	*F*_(1, 56)_ = 0.11	0.746	< 0.01
	Housing x Time	F_(1, 56)_ = 13.13	0.001*	0.19
	ToD x Time	F_(1, 56)_ = 0.70	0.407	0.01
	Sex x Housing x ToD	*F*_(1, 56)_ = 1.84	0.180	0.03
	Sex x Housing x Time	F_(1, 56)_ = 0.47	0.498	0.01
	Sex x ToD x Time	F_(1, 56)_ = 4.51	0.038*	0.08
	Housing x ToD x Time	F_(1, 56)_ = 2.12	0.151	0.04
	Sex x Housing x ToD x Time	F_(1, 56)_ = 0.01	0.944	< 0.01
Total time with nose in closed door	Sex	*F*_(1, 45)_ = 9.40	0.004*	0.17
	Housing	*F*_(1, 45)_ < 0.01	0.997	< 0.01
	ToD	*F*_(1, 45)_ = 0.14	0.711	< 0.01
	Session	F_(5.474, 246.319)_ = 2.01	0.072	0.04
	Sex x Housing	*F*_(1, 45)_ = 0.41	0.524	0.01
	Housing: Females^a^	*F*_(1, 45)_ = 0.21	0.653	0.01
	Housing: Males^a^	*F*_(1, 45)_ = 0.21	0.652	0.01
	Sex x ToD	*F*_(1, 45)_ < 0.01	0.958	< 0.01
	Sex x Session	F_(5.474, 246.319)_ = 2.95	0.011*	0.06
	Housing x ToD	*F*_(1, 45)_ = 0.85	0.360	0.02
	Housing x Session	F_(5.474, 246.319)_ = 0.78	0.574	0.02
	ToD x Session	F_(5.474, 246.319)_ = 0.45	0.832	0.01
	Sex x Housing x ToD	*F*_(1, 45)_ = 0.09	0.771	< 0.01
	Sex x Housing x Session	F_(5.474, 246.319)_ = 0.49	0.798	0.01
	Sex x ToD x Session	F_(5.474, 246.319)_ = 1.95	0.080	0.04
	Housing x ToD x Session	F_(5.474, 246.319)_ = 0.35	0.899	0.01
	Sex x Housing x ToD x Session	F_(5.474, 246.319)_ = 0.61	0.708	0.01
Proportion of closed-door time with nose in door	Sex	*F*_(1, 45)_ = 0.22	0.638	0.01
	Housing	*F*_(1, 45)_ = 2.32	0.135	0.05
	ToD	*F*_(1, 45)_ = 0.23	0.633	0.01
	Session	F_(7, 315)_ = 2.53	0.015*	0.05
	Sex x Housing	*F*_(1, 45)_ = 0.12	0.733	< 0.01
	Housing: Females^a^	*F*_(1, 45)_ = 1.72	0.197	0.04
	Housing: Males^a^	*F*_(1, 45)_ = 0.71	0.406	0.02
	Sex x ToD	*F*_(1, 45)_ = 0.20	0.654	0.01
	Sex x Session	F_(7, 315)_ = 1.63	0.127	0.04
	Housing x ToD	*F*_(1, 45)_ = 0.63	0.430	0.01
	Housing x Session	F_(7, 315)_ = 0.79	0.598	0.02
	ToD x Session	F_(7, 315)_ = 0.77	0.613	0.02
	Sex x Housing x ToD	*F*_(1, 45)_ = 0.24	0.626	0.01
	Sex x Housing x Session	F_(7, 315)_ = 0.40	0.905	0.01
	Sex x ToD x Session	F_(7, 315)_ = 2.45	0.018*	0.05
	Housing x ToD x Session	F_(7, 315)_ = 0.29	0.956	0.01
	Sex x Housing x ToD x Session	F_(7, 315)_ = 1.01	0.422	0.02

^a^Simple effects contrast

^#^Statistically significant with Bonferroni correction applied to simple effects contrasts (*p* <.025)

Across acquisition sessions, a sex difference was observed for several outcomes; female rats obtained more social rewards (Fig. 2A), exhibited greater active-inactive difference scores (Fig. 2B), shorter latencies to first active lever presses (Fig. 2C), and more locomotor activity (Fig. 2D) than males. Within acquisitions sessions 7 and 8, female rats obtained more social rewards than males (Fig. 2E). Sex differences were also identified for video tracking outcomes: female rats spent a greater total duration of time with their nose in the open door than males (Fig. 2F), and male rats spent more time with their nose in the closed door than female rats (Fig. 2I).

Social housing conditions also influenced various outcomes during acquisition; isolated rats obtained more social rewards and exhibited greater locomotor activity than pair-housed rats. Furthermore, isolated rats spent both more total time and a greater proportion of open-door time with their nose in the open door (Fig. 2G). This pattern was consistent with acquisition sessions 7 and 8 wherein isolation increased mean social rewards and the mean proportion of open-door time rats spent with their nose in door (Fig. 2H), relative to pair-housed rats. Notably, across several outcomes, the influence of housing condition was sex-dependent. The isolation-induced increase in total time with nose in open door and proportion of open-door time with nose in open door—both across sessions and within sessions 7 and 8—was greater for female rats compared to males. Moreover, the isolation-induced increase in social rewards (both across sessions and within sessions 7 and 8), locomotion, total time with nose in open door, and proportion of open-door time with nose in open door (both across sessions and within sessions 7 and 8) was only found for female rats and not males.

Most outcomes varied as a function of session number—social rewards, difference in active-inactive lever presses, latency to first active lever press, locomotion, total time with nose in open door, proportion of open-door time with nose in door, and proportion of closed-door time with nose in door—demonstrating patterns relating to the acquisition of social operant responding. Across sessions, social rewards increased at a decreasing rate (linear [*F*(1, 56) = 12.62, *p* =.001, *η*^*2*^ = 0.18] and quadratic [*F*(1, 56) = 20.57, *p* <.001, *η*^*2*^ = 0.27] trends), the difference in active-inactive lever presses increased at a decreasing rate (linear [*F*(1, 56) = 52.10, *p* <.0001, *η*^2^ = 0.48] and quadratic [*F*(1, 56) = 8.19, *p* =.006, *η*^2^ = 0.13] trends), latency to first active lever press decreased (linear trend [*F*(1, 56) = 85.80, *p* <.0001, *η*^2^ = 0.61]), locomotor activity decreased at a decreasing rate (linear [*F*(1, 45) = 26.71, *p* <.0001, *η*^2^ = 0.37] and quadratic [*F*(1, 45) = 4.40, *p* =.042, *η*^2^ = 0.09] trends), time spent with nose in open door increased at a decreasing rate (quadratic trend [*F*(1, 45) = 6.54, *p* =.014, *η*^2^ = 0.13]), and the proportion of open-door time with nose in door decreased at a decreasing rate (linear [*F*(1, 42) = 15.90, *p* <.001, *η*^2^ = 0.28] and quadratic [*F*(1, 42) = 5.99, *p* =.019, *η*^2^ = 0.13] trends). For several outcomes, the influence of session was dependent on biological sex. The increase in social rewards and active-inactive lever press difference across sessions was greater for female than male rats, the reduction in locomotion over sessions was more pronounced for male than female rats, and the increase in total time with nose in closed door was more marked for male than female rats (see Table 2).

Timepoint within acquisition sessions 7 and 8 also impacted social outcomes (Fig. 2E); over session time course, mean social rewards decreased at a decreasing rate (linear [*F*(1, 56) = 370.56, *p* <.0001, *η*^2^ = 0.87] and quadratic [*F*(1, 56) = 72.50, *p* <.0001, *η*^2^ = 0.56] trends). This temporal pattern differed by biological sex, indicating that the reduction over time was greater for males than females. However, a *Time* x *Sex* x *Housing* interaction revealed that the less pronounced reduction in social rewards for females was primarily driven by the isolated females, who did not reduce acquisition of rewards over the session as markedly as the other conditions. Congruently, rats spent a greater fraction of open-door time with their nose in the door during the first three compared to the last three social rewards obtained. This pattern was dependent on housing condition, where the difference between the first and last three social rewards obtained was greater for isolated than pair-housed rats. Additionally, a significant *Sex* x *ToD* x *Time* interaction was found, indicating that during the light phase, female rats spent a greater portion of open-door time with their nose in the door than male rats during the first three compared to the last three social rewards obtained.

Time-of-day and interactions with ToD only influenced a few behavioural outcomes during the acquisition of social operant conditioning. Rats tested during the light phase spent a greater proportion of open-door time with their nose in the door than rats tested in the dark phase. The reduction in locomotion across consecutive sessions was greater for rats tested in the dark phase than light phase. The increase in the difference between male and females towards the later acquisition sessions in the proportion of closed-door time with nose in the door was greater for rats tested in the light phase compared to the dark phase. However, no other significant main effects of ToD or other interactions with ToD were found (Figure S4).

### ToD switch (Phase 1b)

Given the absence of clear ToD effects on social operant outcomes in Phase 1a, Phase 1b aimed to determine whether circadian entrainment of social operant responding had developed during acquisition. For brevity’s sake, only effects pertaining to ToD and ToD switch will be discussed below; complete results are reported in supplemental Sect. 3 alongside Figure S5 depicting the results from Phase 1b and Table S2 summarising the corresponding statistical analyses.

Switching ToD impacted locomotion: rats exhibited greater locomotor activity post-switch compared to pre-switch (Figure S5G and H). The influence of switching ToD on social rewards and difference in active-inactive lever presses was dependent on the ToD rats were previously tested; rats that experienced delayed testing post-switch (i.e., pre-switch light phase) obtained fewer social rewards and a lower active-inactive difference, whereas rats that experienced advanced testing post-switch (i.e., pre-switch dark phase) achieved more social rewards and a greater active-inactive difference (Figure S5A-D). For social rewards, the impact of switching ToD was sex-dependent, where male rats obtained more social rewards post-switch compared to female rats which obtained fewer social rewards. Furthermore, a significant *Housing* x *ToD* x *Switch* interaction effect was observed; the pattern of lower time spent with nose in open door for rats previously tested under the light phase and higher time spent for rats previously tested under the dark phase was more marked for isolated rats than for pair-housed rats (Figure S5I and J).

### Between-session behavioural economics (Phase 2)

**Fig. 3:**
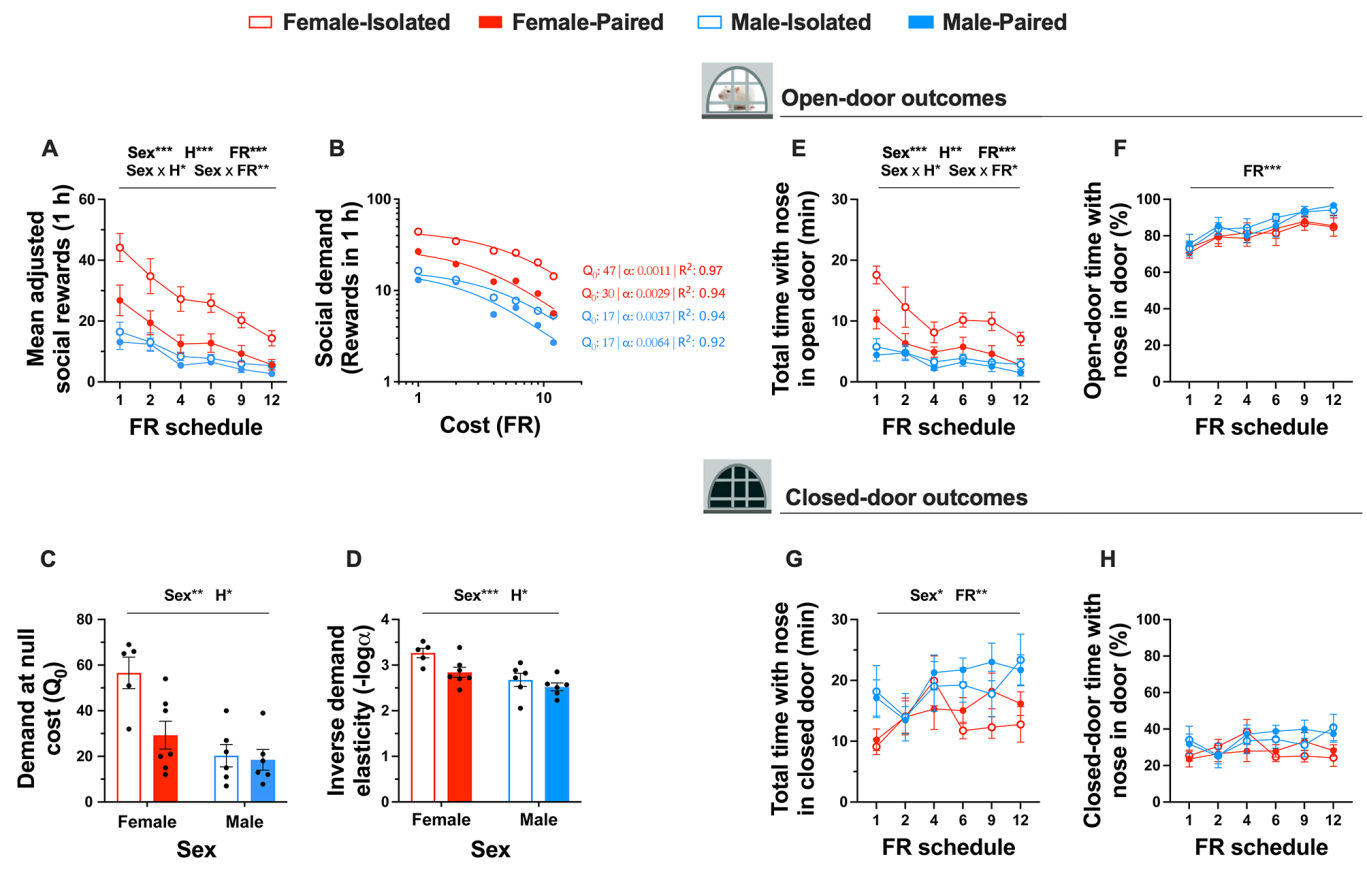
The influence of sex and housing conditions on social operant outcomes from between-session behavioural economics. Effects of biological sex, housing condition, and effort required for social reward on mean adjusted social rewards (**A**); population social demand curves (**B**); social demand at null cost (**C**); social demand elasticity (**D**); total time with nose in open door (**E**); proportion of open door time with nose in open door (**F**); total time with nose in closed door (**G**); and proportion of closed door time with nose in closed door (**H**). Sample sizes are detailed as follows: population demand curves (*n* = 8 per factorial condition); social demand at null cost and social demand elasticity, (F-Iso: *n* = 6, F-Pair: *n* = 7, M-Iso: *n* = 6, M-Pair: *n* = 6); total time with nose in open door, total time with nose in closed door, and proportion of closed door time with nose in closed door (F-Iso: *n* = 8, F-Pair: *n* = 7, M-Iso: *n* = 8, M-Pair: *n* = 7); proportion of open door time with nose in open door (F-Iso: *n* = 8, F-Pair: *n* = 6, M-Iso: *n* = 7, M-Pair: *n* = 5). Note that demand elasticity data was log transformed for analysis and inverted for ease of visualisation. Data represent mean values ± S.E.M and data points represent individual subject data. Statistical significance is indicated by the following: Sex– biological sex; H– housing condition; FR– FR schedule. Level of statistical significance is indicated by the number of * symbols: one– *p* <.05, two– *p* <.01, three– *p* <.001

In Phase 2, we sought to assess the effects of biological sex, housing conditions, and effort required for social reward on social motivation and related outcomes by applying a between-session behavioural economics procedure to social operant conditioning. Figure 3 illustrates the results and Table 3 summarises the corresponding statistical analyses from Phase 2.

**Table 3:** Summary of statistical analyses for Phase 2 (Between-session behavioural economics)

Outcome	Effect	F statistic	p-value	Partial η^2^
*Between-session behavioural economics (Phase 2)*				
Mean adjusted social rewards	Sex	*F*_(1, 28)_ = 26.71	< 0.001*	0.49
	Housing	*F*_(1, 28)_ = 10.06	< 0.001*	0.26
	FR schedule	*F*_(2.061, 57.721)_ = 61.45	< 0.001*	0.69
	Sex x Housing	*F*_(1, 28)_ = 5.30	0.029*	0.16
	Housing: Females^a^	*F*_(1, 28)_ = 14.98	0.001^#^	0.35
	Housing: Males^a^	*F*_(1, 28)_ = 0.38	0.544	0.01
	Sex x FR schedule	*F*_(2.061, 57.721)_ = 8.42	0.001*	0.23
	Housing x FR schedule	*F*_(2.061, 57.721)_ = 1.00	0.376	0.04
	Sex x Housing x FR schedule	*F*_(2.061, 57.721)_ = 0.88	0.422	0.03
Demand at null cost (Q_0_)	Sex	*F*_(1, 21)_ = 15.00	0.001*	0.42
	Housing	*F*_(1, 21)_ = 5.25	0.032*	0.20
	Sex x Housing	*F*_(1, 21)_ = 3.84	0.063	0.16
	Housing: Females^a^	*F*_(1, 21)_ = 9.38	0.006^#^	0.31
	Housing: Males^a^	*F*_(1, 21)_ = 0.05	0.821	< 0.01
Demand elasticity (logα)	Sex	*F*_(1, 21)_ = 18.25	< 0.001*	0.47
	Housing	*F*_(1, 21)_ = 7.82	0.011*	0.27
	Sex x Housing	*F*_(1, 21)_ = 2.00	0.172	0.09
	Housing: Females^a^	*F*_(1, 21)_ = 9.21	0.006^#^	0.31
	Housing: Males^a^	*F*_(1, 21)_ = 0.92	0.349	0.04
Total time with nose in open door	Sex	*F*_(1, 26)_ = 24.26	< 0.001*	0.48
	Housing	*F*_(1, 26)_ = 8.10	0.009*	0.24
	FR schedule	*F*_(2.893, 75.216)_ = 16.07	< 0.001*	0.38
	Sex x Housing	*F*_(1, 26)_ = 4.75	0.039*	0.15
	Housing: Females^a^	*F*_(1, 26)_ = 12.62	0.001^#^	0.33
	Housing: Males^a^	*F*_(1, 26)_ = 0.22	0.641	0.01
	Sex x FR schedule	*F*_(2.893, 75.216)_ = 3.73	0.016*	0.13
	Housing x FR schedule	*F*_(2.893, 75.216)_ = 0.59	0.619	0.02
	Sex x Housing x FR schedule	*F*_(2.893, 75.216)_ = 0.66	0.577	0.03
Proportion of open-door time with nose in door	Sex	*F*_(1, 22)_ = 1.95	0.176	0.08
	Housing	*F*_(1, 22)_ = 0.01	0.930	< 0.01
	FR schedule	*F*_(5, 110)_ = 15.29	< 0.001*	0.41
	Sex x Housing	*F*_(5, 110)_ < 0.01	0.981	< 0.01
	Housing: Females^a^	*F*_(1, 22)_ < 0.01	0.963	< 0.01
	Housing: Males^a^	*F*_(1, 22)_ = 0.01	0.939	< 0.01
	Sex x FR schedule	*F*_(5, 110)_ = 0.30	0.913	0.01
	Housing x FR schedule	*F*_(5, 110)_ = 0.49	0.783	0.02
	Sex x Housing x FR schedule	*F*_(5, 110)_ = 0.31	0.905	0.01
Total time with nose in closed door	Sex	*F*_(1, 26)_ = 6.46	0.017*	0.20
	Housing	*F*_(1, 26)_ = 0.16	0.696	0.01
	FR schedule	*F*_(5, 130)_ = 3.52	0.005*	0.12
	Sex x Housing	*F*_(1, 26)_ = 0.01	0.916	< 0.01
	Housing: Females^a^	*F*_(1, 26)_ = 0.04	0.840	< 0.01
	Housing: Males^a^	*F*_(1, 26)_ = 0.13	0.726	0.01
	Sex x FR schedule	*F*_(5, 130)_ = 1.62	0.160	0.06
	Housing x FR schedule	*F*_(5, 130)_ = 0.79	0.556	0.03
	Sex x Housing x FR schedule	*F*_(5, 130)_ = 1.13	0.348	0.04
Proportion of closed-door time with nose in door	Sex	*F*_(1, 26)_ = 3.71	0.065	0.13
	Housing	*F*_(1, 26)_ < 0.01	0.991	< 0.01
	FR schedule	*F*_(5, 130)_ = 1.57	0.174	0.06
	Sex x Housing	*F*_(1, 26)_ = 0.24	0.626	0.01
	Housing: Females^a^	*F*_(1, 26)_ = 0.12	0.736	< 0.01
	Housing: Males^a^	*F*_(1, 26)_ = 0.13	0.724	0.01
	Sex x FR schedule	*F*_(5, 130)_ = 1.52	0.189	0.06
	Housing x FR schedule	*F*_(5, 130)_ = 0.80	0.552	0.03
	Sex x Housing x FR schedule	*F*_(5, 130)_ = 1.13	0.348	0.04

^a^Simple effects contrast

^#^Statistically significant with Bonferroni correction applied to simple effects contrasts (*p* <.025)

Application of the exponential demand equation [97] to social reward data resulted in demand curves that fit the data with a mean R^2^ of 0.87 (range: 0.64–0.99), following the exclusion of R^2^ values < 0.6. Population social demand curves, representing the average data for each Sex x Housing condition, are illustrated in Fig. 3B. Demand at null cost and demand elasticity were negatively correlated [*r*(23) = − 0.618, *p* =.001] (Figure S6B). When this relationship was examined separately by sex, the significant negative association between Q_0_ and α appeared to be primarily driven by female rats [*r*(10) = − 0.782, *p* =.003] and not male rats [*r*(11) = − 0.515, *p* =.266], although these correlations did not significantly differ by sex (*z* = -1.05, *p* =.294). Social isolation-dependent sex differences were observed across both behavioural economics outcomes. On average, female rats demonstrated a higher preferred level of social reward at null cost (Q_0_) and reduced demand elasticity (i.e., lower sensitivity to increased cost, indicating higher motivation) compared to males (Fig. 3C), and isolated rats exhibited higher average Q_0_ values and reduced demand elasticity for social reward compared to pair-housed rats. However, as this isolation-induced increase in Q_0_ and reduction in demand elasticity was only observed for female and not male rats, it is likely that both main effects of *Sex* and *Housing* are driven by their interaction: a female-specific isolation-induced enhancement of social motivation.

Isolation-dependent sex differences were also observed for non-behavioural economics outcomes; averaged over FR schedules, female rats obtained more social rewards (Fig. 3A), spent more time with their nose in the open door (Fig. 3E), and spent less time with their nose in the closed door (Fig. 3G) than male rats. Isolated rats achieved more social rewards and spent a greater duration of time with their nose in the open door than pair-housed rats. However, this influence of housing was sex-dependent: the isolation-induced increase in social rewards and time spent with nose in the open door was both greater for female rats than males and only observed in females and not males.

The effort required to earn a social reward (i.e., FR schedule) also impacted various outcomes. Across the pattern of increasing FR schedules, the number of social rewards obtained decreased at a decreasing rate (linear [*F*(1, 28) = 92.75, *p* <.001, *η*^2^ = 0.77] and quadratic [*F*(1, 28) = 20.21, *p* <.001, *η*^2^ = 0.42] trends), total time spent with their nose in the open door decreased at a decreasing rate (linear [*F*(1, 26) = 39.90, *p* <.001, *η*^2^ = 0.61] and quadratic [*F*(1, 26) = 8.84, *p* =.006, *η*^2^ = 0.25] trends), the proportion of open-door time rats spent with their nose in the door increased (linear trend [ *F*(1, 22) = 98.93, *p* <.001, *η*^2^ = 0.82]) and the total time spent with their nose in the closed door increased (linear trend [*F*(1, 26) = 6.85, *p* =.015, *η*^2^ = 0.21]). Further, this effect of FR schedule was sex-dependent—the reduction in social rewards obtained and time spent with nose in the open door with increasing FR schedule was greater for female than male rats.

### Stimulus partner sex and within-session behavioural economics (Phase 3)

**Fig. 4:**
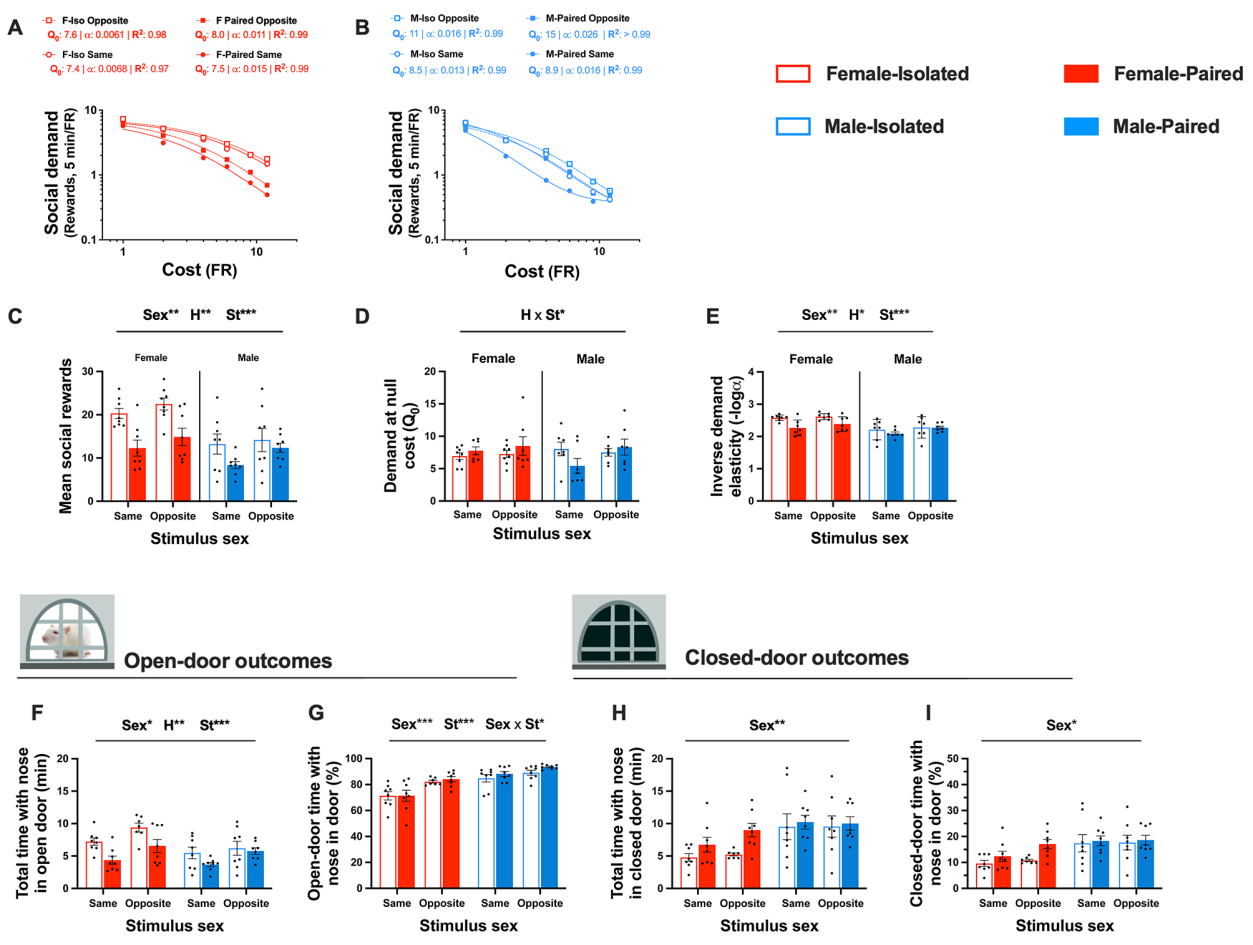
The influence of sex, housing, and stimulus sex on social operant outcomes from within-session behavioural economics. Effects of biological sex, housing conditions, and stimulus partner sex on population social demand curves for females (**A**) and males (**B**), mean adjusted social rewards (**C**), social demand at null cost (**D**), social demand elasticity (**E**), total time with nose in open door (**F**), proportion of open-door time with nose in door (**G**), total time with nose in closed door (**H**), and proportion of closed-door time with nose in door (**I**). Sample sizes are detailed as follows: mean social rewards and population demand curves (*n* = 8 per factorial condition); social demand at null cost and social demand elasticity (F-Iso: *n* = 8, F-Pair: *n* = 7, M-Iso: *n* = 7, M-Pair: *n* = 7); total time with nose in open door, proportion of open-door time with nose in door, total time with nose in closed door, and proportion of closed-door time with nose in door (F-Iso: *n* = 7, F-Pair: *n* = 8, M-Iso: *n* = 8, M-Pair: *n* = 8). Note that demand elasticity data was log transformed for analysis and inverted for ease of visualisation. Data represent mean values ± S.E.M and data points represent individual subject data. Statistical significance is indicated by the following: Sex– biological sex; H– housing condition; St– Stimulus sex. Level of statistical significance is indicated by the number of * symbols: one– *p* <.05, two– *p* <.01, three– *p* <.001

**Fig. 5:**
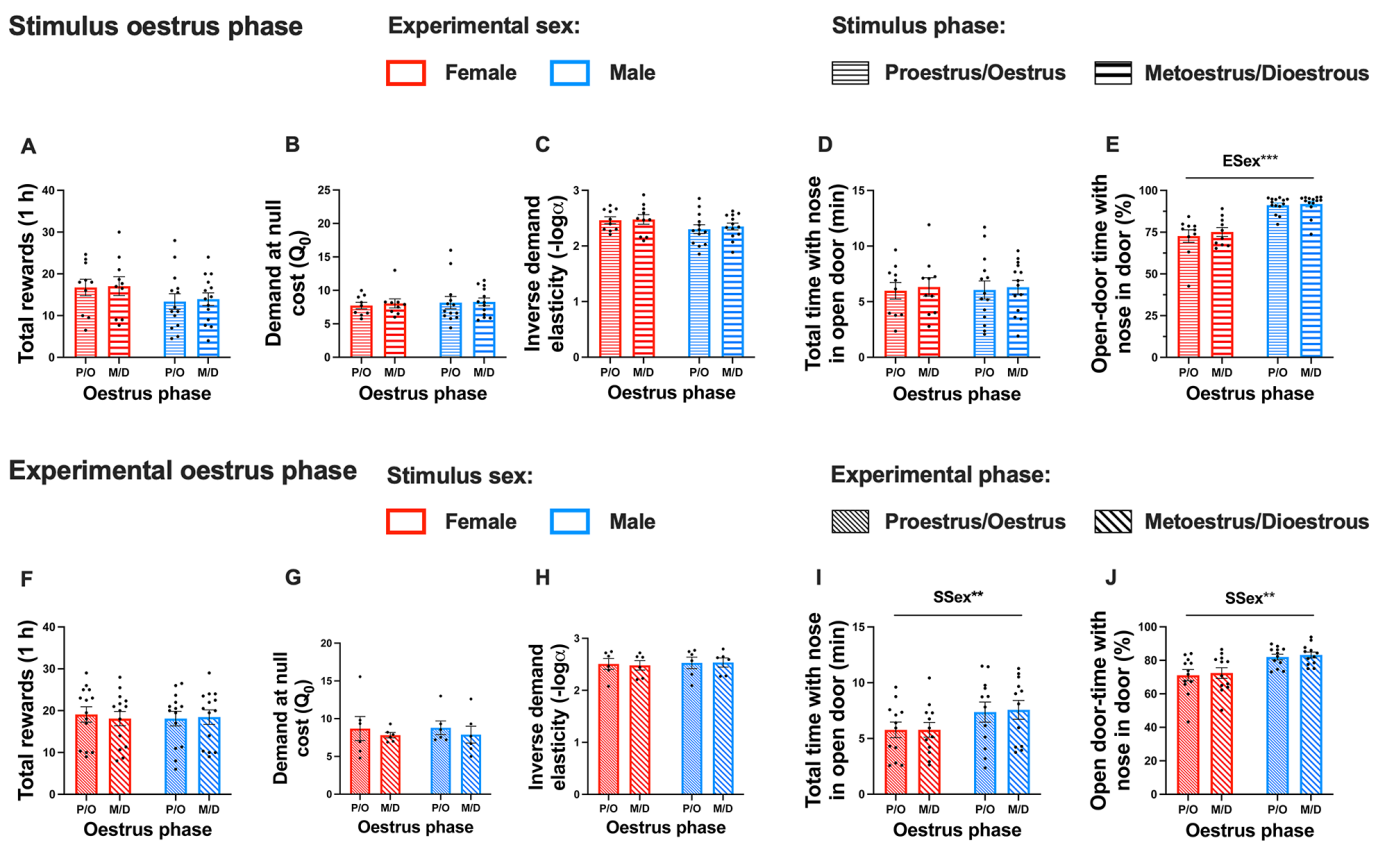
The effect of stimulus and experimental oestrus phase on social operant outcomes from within-session behavioural economics. Effects of stimulus rat oestrus phase on mean total social rewards (**A**), social demand at null cost (**B**), social demand elasticity (**C**), total time with nose in open door (**D**), and proportion of open-door time with nose in door (**E**). The influence of experimental rat oestrus phase on mean total social rewards (**F**), social demand at null cost (**G**), social demand elasticity (**H**), total time with nose in open door (**I**), and proportion of open-door time with nose in door (**J**). Sample sizes are detailed as follows: stimulus phase on mean social rewards, total time with nose in open door, and proportion of open door time with nose in open door (F-P/O: *n* = 10, F-M/D: *n* = 14; M-P/O: *n* = 10, M-P/O: *n* = 14); stimulus phase on demand at null cost and demand elasticity (F-P/O: *n* = 10, F-M/D: *n* = 13; M-P/O: *n* = 10, M-P/O: *n* = 13); experimental phase on mean social rewards (all *n* = 14); stimulus phase on demand at null cost and demand elasticity (all *n* = 6); and experimental phase on total time with nose in open door and proportion of open-door time with nose in door (all *n* = 12). Note that demand elasticity data was log transformed for analysis and inverted for ease of visualisation. Data represent mean values ± S.E.M and data points represent individual subject data. Statistical significance is indicated by the following: ESex– Experimental rat sex; SSex– Stimulus rat sex. Level of statistical significance is indicated by the number of * symbols: one– *p* <.05, two– *p* <.01, three– *p* <.001

In Phase 3, we aimed to explore the effects of stimulus partner sex, alongside biological sex and housing conditions on social motivation and related outcomes, while mitigating the limitations of between-session procedures, by applying a novel within-session behavioural economics procedure to social operant conditioning. Figure 4 illustrates the main results from Phase 3, Fig. 5 examines the influence of oestrus phase on these social operant outcomes, and Table 4 summarises the corresponding statistical analyses.

**Table 4:** Summary of statistical analyses for Phase 3 (Within-session behavioural economics)

Outcome	Effect	F statistic	p-value	Partial η^2^
*Within-session behavioural economics (Phase 3)*				
Mean social rewards	Sex	*F*_(1, 28)_ = 10.28	0.003*	0.27
	Housing	*F*_(1, 28)_ = 10.70	0.003*	0.28
	Stimulus sex	*F*_(1, 28)_ = 23.71	< 0.001*	0.46
	Sex x Housing	*F*_(1, 28)_ = 1.72	0.200	0.06
	Housing: Females^a^	*F*_(1, 28)_ = 10.50	0.003^#^	0.27
	Housing: Males^a^	*F*_(1, 28)_ = 1.92	0.177	0.06
	Sex x Stimulus sex	*F*_(1, 28)_ = 0.01	0.934	< 0.01
	Housing x Stimulus sex	*F*_(1, 28)_ = 3.05	0.092	0.10
	Sex x Housing x Stimulus sex	*F*_(1, 28)_ = 1.76	0.195	0.06
	***Oestrus phase***			
	*Stimulus phase*			
	Stimulus phase	*F*_(1, 22)_ = 0.45	0.510	0.02
	Experimental sex	*F*_(1, 22)_ = 1.55	0.226	0.07
	Stimulus phase x ESex	*F*_(1, 22)_ = 0.04	0.849	< 0.01
	*Experimental phase (Females)*			
	Experimental phase	*F*_(1, 13)_ = 0.71	0.415	0.05
	Stimulus sex	*F*_(1, 13)_ = 2.68	0.126	0.17
	Experimental phase x SSex	*F*_(1, 13)_ = 1.12	0.308	0.08
Demand at null cost (Q_0_)	Sex	*F*_(1, 25)_ = 0.13	0.724	0.01
	Housing	*F*_(1, 25)_ = 0.01	0.930	< 0.01
	Stimulus sex	*F*_(1, 25)_ = 3.33	0.080	0.12
	Sex x Housing	*F*_(1, 25)_ = 1.45	0.240	0.06
	Housing: Females^a^	*F*_(1, 25)_ = 0.86	0.362	0.03
	Housing: Males^a^	*F*_(1, 25)_ = 0.60	0.445	0.02
	Sex x Stimulus sex	*F*_(1, 25)_ = 0.51	0.480	0.02
	Housing x Stimulus sex	*F*_(1, 25)_ = 4.30	0.049*	0.15
	Sex x Housing x Stimulus sex	*F*_(1, 25)_ = 2.69	0.114	0.10
	***Oestrus phase***			
	*Stimulus phase*			
	Stimulus phase	*F*_(1, 21)_ = 0.87	0.362	0.04
	Experimental sex	*F*_(1, 21)_ = 0.51	0.483	0.02
	Stimulus phase x ESex	*F*_(1, 21)_ = 1.01	0.326	0.05
	*Experimental phase (Females)*			
	Experimental phase	*F*_(1, 5)_ = 0.96	0.372	< 0.01
	Stimulus sex	*F*_(1, 5)_ = 0.01	0.940	0.16
	Experimental Phase x SSex	*F*_(1, 5)_ < 0.01	0.994	< 0.01
Demand elasticity (logα)	Sex	*F*_(1, 25)_ = 8.83	0.006*	0.26
	Housing	*F*_(1, 25)_ = 4.92	0.036*	0.16
	Stimulus sex	*F*_(1, 25)_ = 292.91	< 0.001*	0.92
	Sex x Housing	*F*_(1, 25)_ = 1.81	0.190	0.07
	Housing: Females^a^	*F*_(1, 25)_ = 6.56	0.017^#^	0.21
	Housing: Males^a^	*F*_(1, 25)_ = 0.37	0.550	0.02
	Sex x Stimulus sex	*F*_(1, 25)_ = 0.06	0.814	< 0.01
	Housing x Stimulus sex	*F*_(1, 25)_ = 3.25	0.084	0.12
	Sex x Housing x Stimulus sex	*F*_(1, 25)_ = 0.20	0.660	0.01
	***Oestrus phase***			
	*Stimulus phase*			
	Stimulus phase	*F*_(1, 21)_ = 0.63	0.438	0.03
	Experimental sex	*F*_(1, 21)_ = 2.25	0.148	0.10
	Stimulus phase x ESex	*F*_(1, 21)_ = 0.19	0.671	0.01
	*Experimental phase (Females)*			
	Experimental phase	*F*_(1, 5)_ = 0.08	0.789	0.02
	Stimulus sex	*F*_(1, 5)_ = 2.13	0.204	0.30
	Experimental Phase x SSex	*F*_(1, 5)_ = 0.85	0.399	0.15
Total time spent with nose in open door	Sex	*F*_(1, 27)_ = 5.58	0.026*	0.17
	Housing	*F*_(1, 27)_ = 8.38	0.007*	0.24
	Stimulus sex	*F*_(1, 27)_ = 43.45	< 0.001*	0.62
	Sex x Housing	*F*_(1, 27)_ = 1.77	0.195	0.06
	Housing: Females^a^	*F*_(1, 27)_ = 8.63	0.007^#^	0.24
	Housing: Males^a^	*F*_(1, 27)_ = 1.27	0.270	0.05
	Sex x Stimulus sex	*F*_(1, 27)_ = 1.32	0.261	0.05
	Housing x Stimulus sex	*F*_(1, 27)_ = 2.87	0.102	0.10
	Sex x Housing x Stimulus sex	*F*_(1, 27)_ = 0.92	0.345	0.03
	***Oestrus phase***			
	*Stimulus phase*			
	Stimulus phase	*F*_(1, 22)_ = 0.73	0.401	0.03
	Experimental sex	*F*_(1, 22)_ < 0.01	0.980	< 0.01
	Stimulus phase x ESex	*F*_(1, 22)_ = 0.02	0.886	< 0.01
	*Experimental phase (Females)*			
	Experimental phase	*F*_(1, 11)_ = 0.20	0.663	0.02
	Stimulus sex	*F*_(1, 11)_ = 14.60	0.003*	0.57
	Experimental Phase x SSex	*F*_(1, 13)_ = 0.09	0.770	0.01
Proportion of open-door time with nose in door	Sex	*F*_(1, 27)_ = 29.75	< 0.001*	0.52
	Housing	*F*_(1, 27)_ = 0.85	0.366	0.03
	Stimulus sex	*F*_(1, 27)_ = 34.66	< 0.001*	0.56
	Sex x Housing	*F*_(1, 27)_ = 1.02	0.322	0.04
	Housing: Females^a^	*F*_(1, 27)_ < 0.01	0.951	< 0.01
	Housing: Males^a^	*F*_(1, 27)_ = 1.93	0.176	0.07
	Sex x Stimulus sex	*F*_(1, 27)_ = 4.94	0.035*	0.16
	Housing x Stimulus sex	*F*_(1, 27)_ = 1.03	0.319	0.04
	Sex x Housing x Stimulus sex	*F*_(1, 27)_ = 0.35	0.560	0.01
	***Oestrus phase***			
	*Stimulus phase*			
	Stimulus phase	*F*_(1, 22)_ = 0.96	0.337	0.04
	Experimental sex	*F*_(1, 22)_ = 37.83	< 0.001*	0.63
	Stimulus phase x ESex	*F*_(1, 22)_ = 0.30	0.589	0.01
	*Experimental phase (Females)*			
	Experimental phase	*F*_(1, 11)_ = 0.71	0.418	0.06
	Stimulus sex	*F*_(1, 11)_ = 14.91	0.003*	0.58
	Experimental Phase x SSex	*F*_(1, 11)_ < 0.01	0.986	< 0.01
Total time with nose in closed door	Sex	*F*_(1, 27)_ = 7.99	0.009*	0.23
	Housing	*F*_(1, 27)_ = 1.95	0.174	0.07
	Stimulus sex	*F*_(1, 27)_ = 1.59	0.219	0.06
	Sex x Housing	*F*_(1, 27)_ = 0.80	0.380	0.03
	Housing: Females^a^	*F*_(1, 27)_ = 2.53	0.123	0.09
	Housing: Males^a^	*F*_(1, 27)_ = 0.13	0.720	0.01
	Sex x Stimulus sex	*F*_(1, 27)_ = 2.13	0.156	0.07
	Housing x Stimulus sex	*F*_(1, 27)_ = 1.30	0.265	0.05
	Sex x Housing x Stimulus sex	*F*_(1, 27)_ = 1.98	0.171	0.07
Proportion of closed-door time with nose in door	Sex	*F*_(1, 27)_ = 7.09	0.013*	0.21
	Housing	*F*_(1, 27)_ = 1.60	0.217	0.06
	Stimulus sex	*F*_(1, 27)_ = 3.43	0.075	0.11
	Sex x Housing	*F*_(1, 27)_ = 0.65	0.429	0.02
	Housing: Females^a^	*F*_(1, 27)_ = 2.06	0.162	0.07
	Housing: Males^a^	*F*_(1, 27)_ = 0.11	0.743	< 0.01
	Sex x Stimulus sex	*F*_(1, 27)_ = 2.26	0.144	0.08
	Housing x Stimulus sex	*F*_(1, 27)_ = 2.05	0.164	0.07
	Sex x Housing x Stimulus sex	*F*_(1, 27)_ = 1.96	0.173	0.07

^a^Simple effects contrast

^#^Statistically significant with Bonferroni correction applied to simple effects contrasts (*p* <.025)

Application of the exponential demand equation to social reward data generated demand curves that fit the data with a mean R^2^ of 0.88 (range: 0.62–0.99), following the exclusion of R^2^ values < 0.6. Population social demand curves, representing the average data for each *Sex* x *Housing* x *Stimulus sex* condition, are illustrated for female and male rats in Fig. 4A and B, respectively. Social demand at null cost and social demand elasticity were not significantly associated [*r*(27) = − 0.300, *p* =.114] (Figure S6C). Social demand at null cost from between- and within- session behavioural economics (Phases 2 and 3, respectively) were also not significantly associated [*r*(19) = 0.159, *p* =.492] (Figure S6A). In contrast, social demand elasticity from between- and within-session behavioural economics were strongly positively correlated [*r*(19) = 0.829, *p* <.001] (Figure S6D).

The biological sex of stimulus partners influenced several social operant outcomes during within-session behavioural economics procedures. Compared to rats rewarded with a same-sex stimulus, those rewarded with an opposite-sex stimulus partner obtained more social rewards (Fig. 4C), demonstrated reduced demand elasticity (i.e., higher motivation) (Fig. 4E), spent more total time with their nose in the open door (Fig. 4F), and spent a greater proportion of open-door time with their nose in the door (Fig. 4G). This opposite-sex stimulus driven increase in the proportion of nose in open door time was greater for female than male rats. Further, a *Housing* x *Stimulus sex* interaction was observed whereby the increased social demand at null cost for opposite-sex social reward was greater for pair-housed than isolated rats (Fig. 4D).

Sex differences were observed across most outcomes: on average, female rats obtained more social rewards, exhibited reduced sensitivity to the cost of social reward, spent greater total time with their nose in the open door, spent less time with their nose in the closed door (Fig. 4H), and spent a lower proportion of open-door and closed-door time with their nose in the door (Fig. 4I), relative to male rats. Additionally, isolated rats achieved more social rewards, demonstrated less social demand elasticity, and spent more time with their nose in the open door compared to pair-housed rats, however these isolation-induced increases were only observed for female and not male rats.

No effects of stimulus rat oestrus phase or interactions with oestrus phase were found for any social operant outcomes tested (Fig. 5A-E). Similarly, no effects of experimental rat oestrus phase or interactions with oestrus phase were observed across all outcomes assessed (Fig. 5F-J).

## Discussion

The current study investigated the influence of common experimental parameters—biological sex, housing conditions, and ToD—and their interactions on the acquisition of social operant conditioning. Biological sex, housing condition, and especially their interaction, were influential model parameters, while ToD did not emerge as a critical factor. Circadian entrainment of social operant responding developed during acquisition; delay of social operant testing appeared to reduce responding, whereas advancement increased responding. Behavioural economics (both between- and, importantly, within-session paradigms) can be applied to the social operant conditioning model to assess social motivation. Regardless of sex, rats demonstrated higher motivation for interaction with an opposite-sex stimulus compared to a same-sex stimulus partner. Lastly, the oestrus phase of female experimental and stimulus rats did not appear to impact social motivation within the social operant model.

### Sex differences and sex-dependent social homeostasis

Across almost all outcomes and experimental phases, biological sex exerted the most robust and potent influences of all experimental parameters on outcomes of social motivation within the social operant conditioning model. Female rats were consistently found to exhibit greater social motivation than male rats, which is in stark contrast to the prevailing evidence from previous studies using the social operant conditioning model, which have mostly identified an absence of sex differences in social outcomes [31–34, 36, 47]. Similarly, in studies using non-operant assays of social interaction and social place preference conditioning in rats, typically no sex difference is identified [98, 99] or males demonstrate greater social interaction or preference than females for same-sex conspecifics [100–103]. Although Marchant et al. [30] did not report whether a sex difference was observed in social rewards earned during acquisition of social self-administration, video analyses during social operant conditioning revealed that female rats spent a greater proportion of open-door periods interacting with stimulus partners than male rats. While the current study did not replicate this specific finding, it does align with our overall result that female rats were more socially inclined than male rats. Chow et al. [32] found no main effect of sex on social rewards obtained but several interaction effects of other variables with sex either reached or almost reached statistical significance (e.g., *p* =.06). Ramsey et al. [43] found that only female mice were able to acquire social operant responding, potentially indicating that males experience insufficient reward from social interaction to reinforce and motivate acquisition of operant responding. However, in a more recent study, these authors demonstrated social operant responding in both female and male CD1 mice, identifying no evident sex differences in social motivational outcomes [104]. Taken together, our finding of higher social motivation in female rats stands in opposition to most prior social operant and non-operant literature.

As predicted, socially isolated housing conditions produced elevations in social motivation compared to paired housing conditions. This corroborates evidence from previous social operant studies demonstrating that isolated housing enhances social motivation [31, 32, 43, 48, 49, 105]. This finding also contributes to the evidence-base for social homeostasis—a system wherein individuals are sensitive to deviations in the quantity and/or quality of social interaction from an optimal set-point [15, 65]. Our observed isolated housing-induced increase in social motivation aligns with rebound increases in social interaction and affiliative behaviour following social deficits experienced during acute social isolation [65]. However, as prior studies investigating the impact of housing in social operant conditioning have only been conducted using single-sex cohorts or small sample mixed-sex cohorts underpowered to detect sex differences, the current study highlights the novel pronounced interaction between biological sex and housing conditions: social isolation-dependent sex differences in social motivation. That is, isolation-induced enhancement of social motivation was more marked or only observed for female rats. Thus, sex differences may exist in circuitry responsible for maintaining social homeostasis; it is possible that female rats are more sensitive to social deficits than males, or that females enact stronger compensatory responses to rectify social deficits than males.

Most previous social operant studies identifying a lack of sex differences in social motivation have been conducted using Sprague-Dawley or Long-Evans rats; hence, it is possible that the observed sex difference in social motivation is specific to Wistar rats, a strain relatively more prone to anxiety-like behaviours [106–108]. However, Augier et al. [31] found no evidence of a sex difference in social self-administration using a similar sample size of Wistar rats as the current study, although these rats were all group-housed unlike the current study. Taken together, it is plausible the observed sex difference was primarily driven by a strain-specific interaction between biological sex and housing condition. That is, isolation-induced elevations in social motivation for female but not male Wistar rats may have amplified the overall sex difference.

### Stimulus partner sex and oestrus phase

Surprisingly, both female and male rats demonstrated higher motivation for opposite-sex stimulus partners than same-sex stimulus partners. This stands contrary to the only previous social operant study to examine the influence of stimulus sex on both male and female subjects, which identified an opposite-sex stimulus partner preference solely for male and not female rats [64]. However, the current finding does align with other previous rodent studies of preferences for opposite-sex conspecifics in male [55, 109] and female [80, 110] rats. A notable limitation of the same- vs. opposite-sex stimulus comparison from Phase 3 is that opposite-sex stimuli were completely novel to experimental rats whereas same-stimuli stimuli had been previously encountered, although stimulus cycling maintained an interval of seven sessions (i.e., 16 days) between each encounter. Furthermore, the order of exposure to same- vs. opposite-sex stimuli was not conducted as a counterbalanced crossover design. Consequently, the large magnitude effect of stimulus sex on social motivation observed may be conflated by an effect of social novelty and an order of presentation effect.

The rationale behind using same-sex stimulus partners in social operant conditioning likely stems from an intention to assess general social motivation and not sexual motivation [111]. In fact, in one of the only social operant studies to use opposite-sex stimuli for female experimental rats, male stimulus rats were castrated for the explicit purpose of investigating “social, rather than sexual, reinforcement” [42, p155]. However, this experimentally manipulated distinction between social and sexual motivation may not be as straightforward as proposed. Firstly, a previous social operant study demonstrated no difference in motivation of ovariectomised female mice between those with and without oestradiol replacement for opposite-sex stimuli [49], despite oestradiol replacement strongly increasing lordosis (i.e., sexual receptivity). This suggests that female mice with and without sexual drive demonstrate approximately equivalent motivation within social operant conditioning. Secondly, numerous rodent studies have found that partner sex preferences can be shifted from opposite-sex to same-sex through environmental and endocrine manipulations [55, 110, 112–115], meaning that sexual motivation may not be exclusive to opposite-sex stimuli. Lastly, if access to a same- vs. opposite-sex stimulus distinctly engage social and sexual motivation, respectively, and if social and sexual motivation are distinct constructs, it would be expected that an association between measures of motivation for same- vs. opposite-sex stimuli should be relatively weak or negligible. However, values of demand elasticity (α) for same- and opposite-sex stimuli from the current study were significantly and very strongly associated (*r*_(27)_ = 0.897, see supplemental Sect. 5.3 and Table S5 for further details). Taken together, this may suggest that responding for same- vs. opposite-sex stimuli do not necessarily engage distinct motivational constructs, and that motivation for opposite-sex interaction may predominantly reflect social motivation within the social operant conditioning model.

No influence of experimental or stimulus rat oestrus phase on any primary outcomes from social operant conditioning was observed in the current study. This was unexpected given the potent control that the oestrus cycle exhibits over female sexual behaviour and consequently, the behaviour of males toward females [116]. This finding stands contrary to other research which highlight an influence of the oestrus cycle on social interaction in female mice [117], preference of partner sex in female rats [118], social behaviour in polygynous *Peromyscus* mice [119]. However, this result does align with some previous studies that identified no effect of oestrus phase on social motivation in the social operant model [64], and social interaction with a same-sex conspecific in an approach-avoidance task [98]. It is possible that the social operant model employed in the current study does not offer the full-contact and unrestricted mobility—whereby females can express proceptive behaviours [120]—necessary for the influence of oestrus phase on social and/or sexual motivation to manifest.

### The social operant conditioning model: practical and theoretical considerations

For a summary of methodological recommendations for conducting social operant conditioning to best assess social motivation based on the current study, refer to supplemental Sect. 6.

Given that social operant conditioning utilises access to social interaction as a reinforcer, and since rats exhibit a broad and complex repertoire of social behaviours during interactions [121], ‘consumption’ of the operant reward within this model is—likewise—complex. While most previous studies using this model have relied on lever press-derived outcomes to assess appetitive behaviours indicative of social motivation, they have neglected the potential complexity of social interaction during reward delivery. Using video recording, pose estimation, and kinematic analyses alongside conventional operant assessment, the current study confirms that while appetitive outcomes do align with some consummatory outcomes, this was not consistently the case. For example, while the effects of experimental parameters on total time rats spent with their noses in the open door matched closely to social rewards across all experimental phases, this was not the case for the proportion of open-door time rats spent with noses in the door. In fact, when significant associations between social rewards and the proportion of open-door time spent in social investigation were found, these were predominantly negative, potentially indicating that more social rewards lead to satiety of social interaction over the course of a 1-h session. This pattern of associations with proportion of open-door time was also found for behavioural economics indicators of motivation (see supplemental material Sect. 5.4 and 5.5, and Tables S6 and S7). This ‘social satiety’ interpretation aligns with our Phase 1a findings of reductions in proportions of nose in door during open-door time between the first-three and final-three rewards received and front-loading of social rewards within-session. Notably, this front-loading phenomenon is in contrast with recent research by Augier et al. [31], which found equal spacing of social rewards within sessions; this discrepancy may be due to 30-min session duration compared to the 1-h sessions in the current study, so social satiety may not have been achieved within the shorter timeframe. This interpretation of social satiety holds important implications for designing future social operant conditioning experiments. For example, reducing social satiety via shorter session duration may improve session-to-session stability in social motivation, and conversely, longer sessions (and analyses of time course thereof) would be beneficial to explore manipulations expected to reduce or enhance satiety. Additionally, given the resistance of isolated female rats to within-session social satiety in the current study, using this combination of experimental parameters should facilitate further investigation into sex differences in social behaviour.

An assessment of behaviour during closed-door time revealed a noteworthy sex difference in total time rats spent with their noses in the closed door. This investigation of the closed door may still represent motivation for social interaction manifesting in a form that may reflect poor operant learning (e.g., reward seeking without goal-directed behaviour) or constitute a form of monitoring behaviour (e.g., checking whether the social partner is still present in the stimulus compartment). Alternatively, closed-door investigation time may reflect an effort-related compromise between easily obtaining a partial social reward (e.g., olfactory stimuli that can permeate the closed door) and the higher effort required to earn access to social interaction. Future research should investigate whether qualitatively different levels of social interaction complexity with stimuli—no contact (i.e., only olfactory), partial contact (i.e., through a grille), or full contact (i.e., no grille)—impact responding within the social operant conditioning model [122]. Further, video analysis of consummatory social interactions within the social operant model should examine the components of social behaviours of both experimental *and* stimulus rats. Clearly, the inclusion of video recording in conjunction with conventional operant behavioural assessment provides much richer insight into the nature of social operant behaviour and social motivation than can be gleaned from solely lever press-derived outcomes [105].

In line with previous literature [37, 40], the present study confirms that both between- and within-session behavioural economics approaches can be applied to social operant conditioning and that social demand is elastic (i.e., sensitive to increases in cost), in a large cohort of female and male rats. The current findings also demonstrate that behavioural economics can more comprehensively assess social motivation beyond reward frequency at FR schedules and breakpoint during progressive ratio schedules, providing insight into *both* individual subjects’ hedonic set-points and persistence in spite of increasing cost. For between-session behavioural economics, outcomes of demand at null cost and demand elasticity were convergent in their indications for social motivation, whereas within-session behavioural economics exhibited a divergence between these outcomes, primarily driven by a loss of sensitivity in Q_0_. This between- and within-session discrepancy in Q_0_ may be due to a ceiling effect created by the 5-min duration/FR schedule bin limit implemented during the within-session paradigm, which was imposed to attenuate potential within-session social satiety that might confound assessment of demand elasticity. Hence, the within-session approach may be more appropriately suited to assess changes in social demand elasticity, whereas the between-session approach may provide better sensitivity to assess effects on social demand at null cost. Despite this limitation, the within-session behavioural economics procedure applied to social operant conditioning represents an ideal model for testing acute manipulations (e.g., pharmacology) and overcomes issues of long duration (i.e., weeks between different price points) encountered during between-session procedures [85].

### Perspectives and significance

The current study established a behavioural model that systematically uncovered novel, large magnitude sex differences in social motivation through interactions of biological sex with social housing conditions. This behavioural platform—the combined application of social operant conditioning, within-session behavioural economics, and video tracking—can now be used in future research to elucidate the neurobiological underpinnings of sex differences in social motivation, interaction, and homeostasis. Dopaminergic activity in the ventral tegmental area (VTA) is involved in social motivation during social operant conditioning [14], and previous studies in both humans and mice found social isolation-induced elevations in social motivation were accompanied by amplified VTA activity [66, 123]. This isolation-induced increase in sociality was mediated by alterations to oxytocinergic neurocircuitry in the paraventricular nucleus of the hypothalamus (PVN): increased density of oxytocinergic neurons and hyperexcitability of oxytocinergic neurons projecting to putative VTA dopaminergic neurons [123]. However, as this study was only conducted in male adolescent mice, the generalisability of findings to female mice is unknown. Similarly, during social operant conditioning, Chow et al. [64] recently demonstrated that male—but not female—rats exhibit higher dopaminergic activity in the nucleus accumbens (NAc) during extension of the opposite-sex social stimulus-associated lever, linking enhanced behavioural responding for opposite-sex partners to NAc dopaminergic activity [64]. Notably, in rats, neurons within the NAc express oxytocin receptors and pharmacological manipulations of these receptors modulate behaviours relevant to social motivation including approach and vigilance [124, 125].

On this basis, it is plausible that sex differences in the endogenous oxytocin system and its behavioural outputs may contribute to the overall sex difference in social motivation and sex differences in the effect of isolation on social motivation. Previous research has identified sex differences in: oxytocin receptor binding density in Wistar rats that was associated with outcomes of social interest [126, 127], the behavioural consequences of activating oxytocin receptor-expressing medial prefrontal cortex interneurons in mice (i.e., activation promoted social interaction in females and anxiety-like behaviour in males) [128, 129], and responses to chronic isolation in prairie voles (i.e., only elevated plasma oxytocin levels and activation of PVN oxytocinergic neurons in females and not males) [130, 131]. Furthermore, since previous studies found that oestrus phase had little influence on sex differences in oxytocin receptor binding [52, 127, 132], this may explain the absence of oestrus phase-induced effects on social motivation observed in the current study. Future research should use mixed-sex cohorts in the social operant conditioning model to investigate the potential role of sex differences in oxytocinergic neurocircuitry for: (1) sex differences in baseline social motivation; (2) sex differences in isolation-induced enhancement of social motivation; and (3) the influence of stimulus partner sex on social motivation.

Another valuable avenue for future research involves using the social operant conditioning paradigm to further explore mechanisms and processes underlying social homeostasis. Dose-response relationships between the quantity of social interaction (i.e., isolation duration) and social motivation could be investigated, revealing the temporal profile of social homeostatic processes (i.e., detection of social deficit and compensatory increase in social interaction). Conversely, an adapted outcome devaluation paradigm (e.g., sensory specific satiety), typically employed for operant conditioning studies using drug and food reward [133, 134], could be applied to investigate the temporality of social satiety following a social surplus. Throughout these experiments, manipulations of neural activity within circuitry involved in social reward and homeostasis would provide further insight for frameworks of social homeostasis [65].

Given overt sex differences during development, age is an additional experimental parameter warranting further investigation. Most previous social operant studies were conducted using adult [135] subjects (i.e., at least P60 at start of experimentation), likely due to social operant protocols involving isolated housing for at least one week prior to testing [29] and the aim of avoiding potential neurodevelopmental impacts of isolation during adolescence [53, 136–139]. While Ramsey et al. [43] found no difference in social operant responding between adolescent (PND 26–28) and adult (PND 70–74) mice, this may not generalise to rats given differences in the social repertoire of mice and rats [140]. Achterberg et al. [44] demonstrated successful social operant conditioning in juvenile (P24) male Wistar rats with progressive ratio breakpoints indicative of high social motivation. Given that male adolescent rats exhibit higher levels of social play and greater social preference than female rats [101, 141], the observed sex differences in social motivation in the current study may be absent or reversed in adolescent subjects. Thus, future research should systematically investigate the interaction between sex and age in rats within social operant conditioning.

The present study provides nuanced characterisation of an increasingly utilised preclinical model that more comprehensively assesses social motivation and its role in psychiatric and psychological disorders. Given the critical role that social support and interaction play in health and wellbeing [3–6], the clinical implications of this research extend far beyond basic social behaviour and neurobiology. The application of social operant conditioning has immense transdiagnostic potential for preclinical investigation of substance use, social dysfunction in autism spectrum disorder, schizophrenia spectrum disorders, and sleep-wake disorders. Additionally, the current study stands in opposition to many previous studies that—by design—are statistically underpowered to detect sex differences. Our findings underscore the importance of continued consideration and inclusion of sex as a biological variable in social operant conditioning studies [142].

## Conclusions

Dysfunctions in social motivation, and social behaviour more broadly, are present across a plethora of psychiatric disorders that can afflict individuals across the human lifespan, from early onset in autism spectrum disorder to later development in neurodegenerative disorders. A comprehensive understanding of social motivation, associated behaviours, and neurobiology is critical to developing treatments and restoring individuals’ social functioning—a fundamental component of people’s quality of life and wellbeing [3–6, 143]. This deeper comprehension in the neurocircuitry of social motivation will rely heavily on validated, well-characterised preclinical models of social motivation. The findings of the current study provide novel insights into common experimental parameters of the social operant conditioning model, identifying critical roles for biological sex, housing condition, and stimulus sex. We also highlight the optimal inclusion of behavioural economics (both between- and within-session paradigms) and video recording and analyses alongside social operant conditioning to better assess social motivation. Lastly, to ensure that translation of preclinical research applies to both male and female individuals, the current study emphasises the vital consideration of sex differences and inclusion of mixed-sex cohorts in studies utilising the social operant conditioning model.

### Electronic supplementary material

Below is the link to the electronic supplementary material.

Supplementary Material 1

## Data Availability

All data included and/or analysed in this study are available upon reasonable request from the corresponding author.
