# Supplementary material for: Sex differences in the social motivation of rats: Insights from social operant conditioning, behavioural economics, and video tracking
Source: Biol Sex Differ. 2024 Jul 19;15:57. doi: 10.1186/s13293-024-00612-4 (PMC11264584; doi:10.1186/s13293-024-00612-4)
Supplement: Supplementary file 1 — Supplementary Material 1 [file 13293_2024_612_MOESM1_ESM.pdf]

SUPPLEMENTAL MATERIAL

Sex differences in the social motivation of rats: Insights from social operant conditioning, behavioural economics, and video tracking

Joel S Raymond, Simone Rehn, Morgan H James, Nicholas A Everett[#], Michael T Bowen[#]

1. Inclusion/exclusion of rats from Phases 2 and 3 based on Phase 1 outcomes (p. 2-8)

Includes Figures S1-3 and Table S1.

2. Acquisition (Phase 1a) – ToD analyses and results (p. 9-11)

Includes Figure S4.

3. ToD Switch (Phase 1b) – ToD switch analyses and results (p. 12-18)

Includes Figure S5 and Table S2.

4. Time-of-day, circadian phase, and entrainment: Discussion and implications (p. 19-20)

5. Supplementary correlational analyses of social operant data (p. 21-28)

5.1. Relationships between behavioural economics outcomes from between- and with-session procedures. (p. 21)

Includes Figure S6.

5.2. Predictive relationships between social operant outcomes from Phase 1 and behavioural economics outcomes from Phases 2 and 3. (p. 22-25)

Includes Tables S3 and S4.

5.3. Relationships between behavioural economics outcomes for same- and opposite-sex stimuli from Phase 3. (p. 26)

Includes Table S5.

5.4. Relationships between social rewards and video-derived open-door outcomes. (p. 26-27)

Includes Table S6.

5.5. Relationships between behavioural economics outcomes and social rewards/video-derived open-door outcomes. (p. 27-28)

Includes Tables S7.

6. A guide to common experimental parameters in social operant conditioning (p. 29-32)

Includes Figure S7.

7. References (p. 33)

1. Inclusion/exclusion of rats from Phases 2 and 3 based on Phase 1 outcomes.

During the transition to Phase 2 from Phase 1, the time-of-day (ToD) variable was removed from the experimental design since little to no impact of ToD was found across outcomes of Phase 1a and 1b. As part of this transition, the number of subjects was halved (from n = 64 to n = 32); inclusion of rats in later phases of the experiment was selected based on identifying the 50% highest responders from each *Sex* x *Housing* x *ToD* factorial group and each operant chamber. This was decided to ensure that the chamber conditions (e.g., location of active levers and associated lights) were held identical between phases. Identification of the top 50% of responders was conducted by computing the mean social rewards across all sessions of Phase 1 and selecting the subjects with the four highest values. The social rewards attained by included and excluded rats across Phase 1 sessions are illustrated in Figure S1, and the differences in mean social rewards across Phase 1 session between included and excluded rats are depicted in Figure S2.

If the primary analyses of social operant outcomes from Phase 1 are conducted only using data of rats included in Phase 2 and 3, a similar pattern for results are found with some exceptions (see Figure S3 and Table S1). Across most outcomes, the *Sex* x *Session* interaction effects no longer reach statistical significance, indicating that the rate of acquisition appears to be less dependent on sex when restricted to included subjects. Notably, while the sex difference between females and males remains robust and significant, the *Sex* x *Housing* interaction effect is less pronounced and significant across fewer outcomes when only included subjects are considered. No main effect or interaction effects with ToD were found for any outcome, which emphasises the negligible impact of ToD within the social operant conditioning model, and further justifies the decision to remove it from the experimental design for Phases 2 and 3.

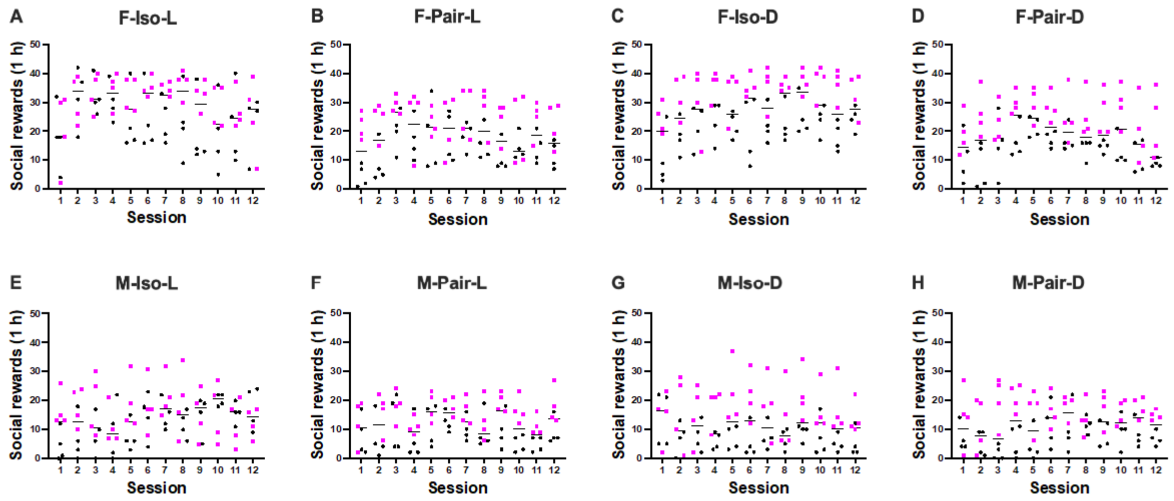

Figure S1. Performance of included and excluded subjects from Phases 2 and 3 during Phase 1a sessions. Social rewards obtained in Phase 1 by included and excluded rats from each Sex x Housing x ToD factorial group. D – dark phase; F – female; Iso – isolated housing; L – light phase; M – male; Pair – paired housing. Data are medians (flat bars), and individual data points represent individual subject data, where colour/shape illustrate inclusion/exclusion in Phases 2 and 3: pink/square – included; black/circle – excluded.

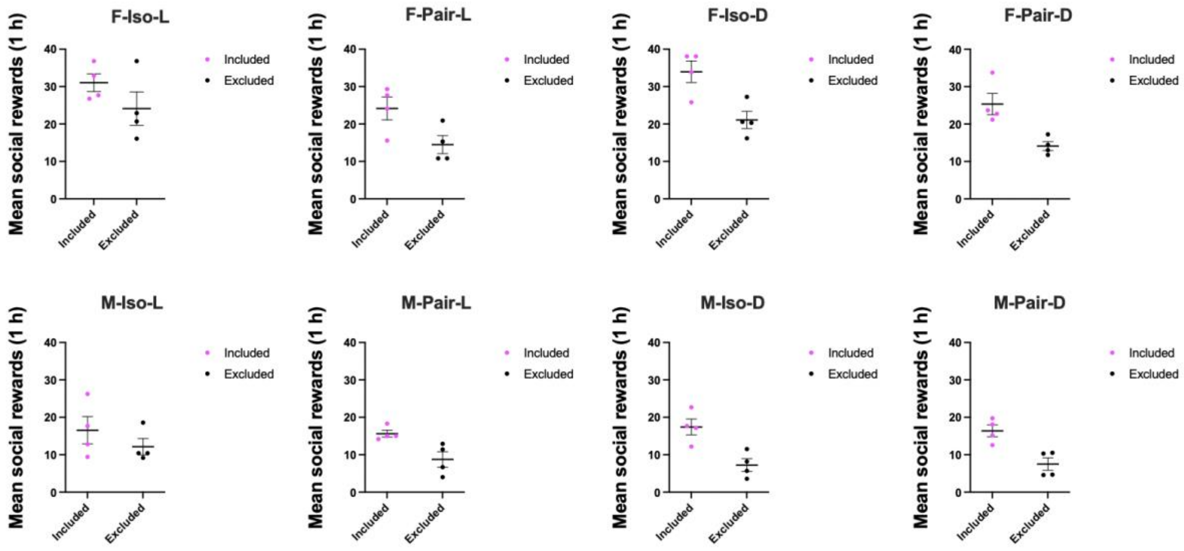

Figure S2. Mean social rewards obtained across Phase 1 sessions by included and excluded rats. Mean social rewards obtained across Phase 1 by included and excluded rats from each Sex x Housing x ToD factorial group. Where excluded rats demonstrate higher responding that some included rats, this was a result of the 'maintaining the same operant chamber' criterion, so if two high performing rats were tested during different runs but using the same chamber, only the highest of these was included in Phases 2 and 3. D – dark phase; F – female; Iso – isolated housing; L – light phase; M – male; Pair – paired housing. Data represent mean ± S.E.M. and individual data points represent individual subject data, where colour/shape illustrate inclusion/exclusion in Phases 2 and 3: pink/square – included; black/circle – excluded.

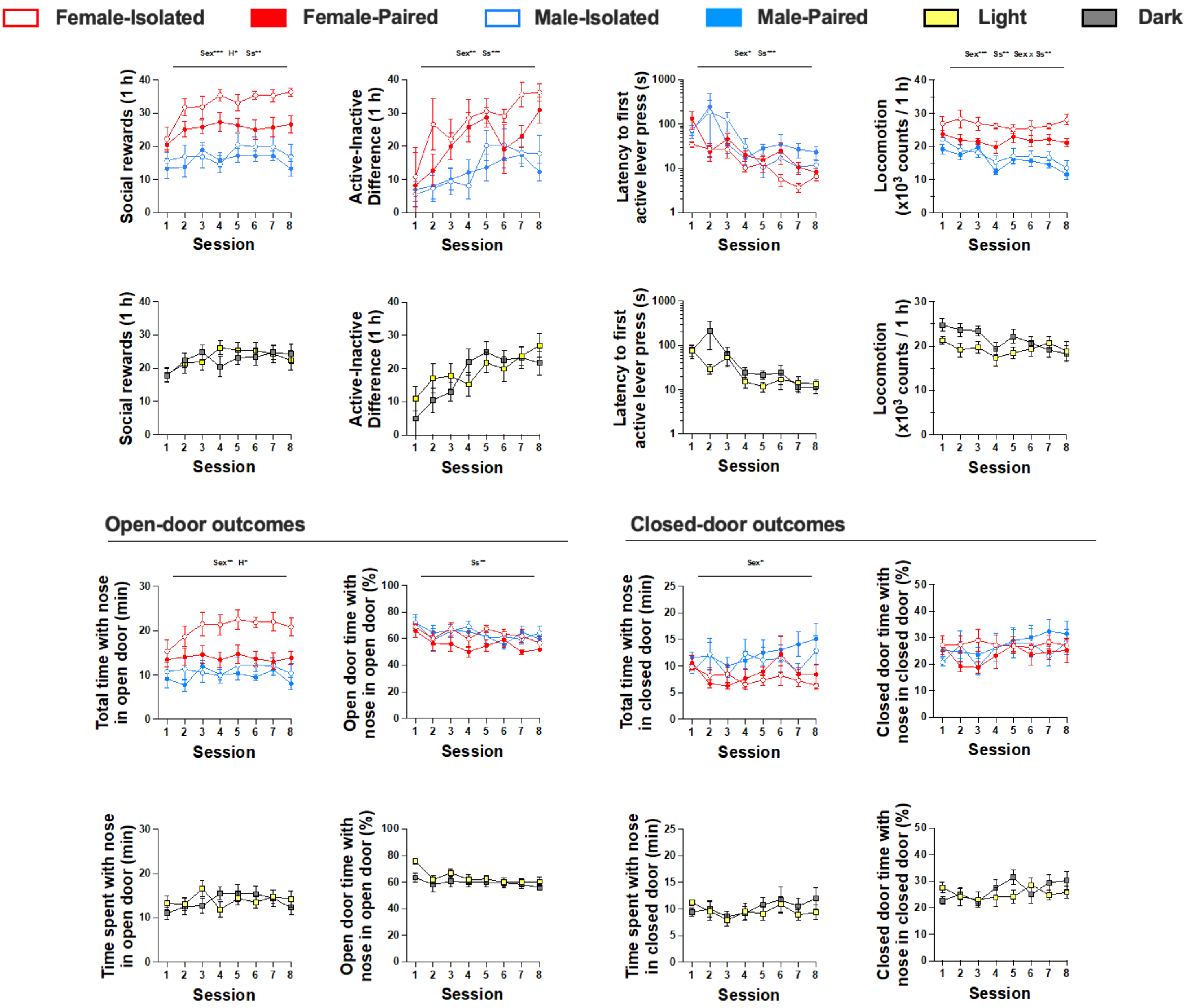

Figure S3. The influence of sex, housing conditions, and time-of-day on outcomes of acquisition in social operant conditioning only in rats included in Phases 2 and 3. Effects of experimental parameters of biological sex, housing conditions, and time-of-day on social rewards, difference in active-inactive lever presses, latency to first active lever press, locomotor activity, total time with nose in open door, proportion of open door time with nose in open door, total time with nose in closed door, and proportion of closed door time with nose in closed door. Data represent mean values ± S.E.M. and statistical significance is indicated by the following: Sex – biological sex; H – housing condition; ToD – time-of-day; Ss – session. Level of statistical significance is indicated by the number of * symbols: one – $p < .05$, two – $p < .01$, three – $p < .001$.

Table S1 – Phase 1a analyses on data from only subjects included in Phases 2 and 3.

Outcome	Effect	*F* statistic	*p*-value	Partial η^2
Acquisition (Phase 1a)				
Social rewards	Sex	$F_{(1, 24)} = 43.74$	< .001*	0.65
	Housing	$F_{(1, 24)} = 6.11$	.021*	0.20
	ToD	$F_{(1, 24)} = 0.09$	.770	< 0.01
	Session	$F_{(3.353, 80.482)} = 4.11$	.007*	0.15
	Session: Linear trend	$F_{(1, 24)} = 4.72$	.040*	0.16
	Session: Quadratic trend	$F_{(1, 24)} = 14.74$	.001*	0.38
	Sex x Housing	$F_{(1, 24)} = 2.23$	.148	0.09
	Housing: Females[a]	$F_{(1, 24)} = 7.86$	.010[#]	0.25
	Housing: Males[a]	$F_{(1, 24)} = 0.48$	.496	0.02
	Sex x ToD	$F_{(1, 24)} = 0.01$	.914	< 0.01
	Sex x Session	$F_{(3.353, 80.482)} = 2.05$	.107	0.08
	Housing x ToD	$F_{(1, 24)} = 0.05$	.828	< 0.01
	Housing x Session	$F_{(3.353, 80.482)} = 0.80$	.510	0.03
	ToD x Session	$F_{(3.353, 80.482)} = 1.63$	.184	0.06
	Sex x Housing x ToD	$F_{(1, 24)} = 0.03$	.867	< 0.01
	Sex x Housing x Session	$F_{(3.353, 80.482)} = 0.56$	.663	0.02
	Sex x ToD x Session	$F_{(3.353, 80.482)} = 0.61$	.626	0.03
	Housing x ToD x Session	$F_{(3.353, 80.482)} = 0.12$	.961	0.01
	Sex x Housing x ToD x Session	$F_{(3.353, 80.482)} = 0.98$	.416	0.04
Difference in active-inactive lever presses	Sex	$F_{(1, 24)} = 10.14$	.004*	0.30
	Housing	$F_{(1, 24)} = 1.12$	.300	0.05
	ToD	$F_{(1, 24)} = 0.09$	.770	< 0.01
	Session	$F_{(3.450, 82.792)} = 10.26$	< .001*	0.30
	Session: Linear trend	$F_{(1, 24)} = 22.50$	< .001*	0.48
	Session: Quadratic trend	$F_{(1, 24)} = 6.41$	.018*	0.21
	Sex x Housing	$F_{(1, 24)} = 0.50$	.485	0.02
	Housing: Females[a]	$F_{(1, 24)} = 1.56$	.223	0.06
	Housing: Males[a]	$F_{(1, 24)} = 0.06$	.807	< 0.01
	Sex x ToD	$F_{(1, 24)} < 0.01$	.988	< 0.01
	Sex x Session	$F_{(3.353, 80.482)} = 1.99$	.114	0.08
	Housing x ToD	$F_{(1, 24)} = 0.28$	.602	0.01
	Housing x Session	$F_{(3.353, 80.482)} = 0.75$	.540	0.03
	ToD x Session	$F_{(3.353, 80.482)} = 1.95$	.119	0.08
	Sex x Housing x ToD	$F_{(1, 24)} = 0.06$	.816	< 0.01
	Sex x Housing x Session	$F_{(3.353, 80.482)} = 0.74$	.548	0.03
	Sex x ToD x Session	$F_{(3.353, 80.482)} = 0.99$	.411	0.04
	Housing x ToD x Session	$F_{(3.353, 80.482)} = 0.25$	.885	0.01

	Sex x Housing x ToD x Session	$F_{(3.353, 80.482)}$ = 1.00	.404	0.04
Latency to active lever first press	Sex	$F_{(1, 24)}$ = 5.10	.033*	0.18
	Housing	$F_{(1, 24)}$ = 2.14	.157	0.08
	ToD	$F_{(1, 24)}$ = 1.01	.325	0.04
	Session	$F_{(4.638, 111.300)}$ = 11.86	< .001*	0.33
	Session: Linear trend	$F_{(1, 24)}$ = 70.58	< .001*	0.75
	Session: Quadratic trend	$F_{(1, 24)}$ = 7.89	.010*	0.25
	Sex x Housing	$F_{(1, 24)}$ = 0.71	.409	0.03
	Housing: Females[a]	$F_{(1, 24)}$ = 2.65	.117	0.10
	Housing: Males[a]	$F_{(1, 24)}$ = 0.19	.664	0.01
	Sex x ToD	$F_{(1, 24)}$ = 0.59	.450	0.02
	Sex x Session	$F_{(4.638, 111.300)}$ = 0.61	.678	0.03
	Housing x ToD	$F_{(1, 24)}$ = 0.01	.910	< 0.01
	Housing x Session	$F_{(4.638, 111.300)}$ = 1.25	.290	0.05
	ToD x Session	$F_{(4.638, 111.300)}$ = 0.67	.637	0.03
	Sex x Housing x ToD	$F_{(1, 24)}$ = 0.18	.673	0.01
	Sex x Housing x Session	$F_{(4.638, 111.300)}$ = 1.03	.403	0.04
	Sex x ToD x Session	$F_{(4.638, 111.300)}$ = 0.25	.930	0.01
	Housing x ToD x Session	$F_{(4.638, 111.300)}$ = 1.16	.326	0.05
	Sex x Housing x ToD x Session	$F_{(4.638, 111.300)}$ = 0.54	.729	0.02
Locomotion	Sex	$F_{(1, 19)}$ = 40.26	< .001*	0.68
	Housing	$F_{(1, 19)}$ = 6.86	.017*	0.27
	ToD	$F_{(1, 19)}$ = 2.95	.102	0.13
	Session	$F_{(5.311, 100.910)}$ = 4.12	.002*	0.18
	Session: Linear trend	$F_{(1, 19)}$ = 12.01	.003*	0.39
	Session: Quadratic trend	$F_{(1, 19)}$ = 0.81	.379	0.04
	Sex x Housing	$F_{(1, 19)}$ = 1.18	.290	0.06
	Housing: Females[a]	$F_{(1, 19)}$ = 6.64	.018[#]	0.26
	Housing: Males[a]	$F_{(1, 19)}$ = 1.21	.285	0.06
	Sex x ToD	$F_{(1, 19)}$ = 0.59	.453	0.03
	Sex x Session	$F_{(5.311, 100.910)}$ = 3.72	.003*	0.16
	Housing x ToD	$F_{(1, 19)}$ = 0.45	.511	0.02
	Housing x Session	$F_{(5.311, 100.910)}$ = 1.32	.258	0.07
	ToD x Session	$F_{(5.311, 100.910)}$ = 1.82	.112	0.09
	Sex x Housing x ToD	$F_{(1, 19)}$ = 1.67	.212	0.08
	Sex x Housing x Session	$F_{(5.311, 100.910)}$ = 0.97	.443	0.05
	Sex x ToD x Session	$F_{(5.311, 100.910)}$ = 1.22	.305	0.06
	Housing x ToD x Session	$F_{(5.311, 100.910)}$ = 1.09	.371	0.05
	Sex x Housing x ToD x Session	$F_{(5.311, 100.910)}$ = 0.86	.516	0.04
	Sex	$F_{(1, 19)}$ = 16.30	.001*	0.46
	Housing	$F_{(1, 19)}$ = 6.98	.016*	0.27

Total time with nose in open door	ToD	$F_{(1, 19)} = 0.01$	.947	< 0.01
	Session	$F_{(3.438, 65.313)} = 1.63$	.184	0.08
	Sex x Housing	$F_{(1, 19)} = 1.97$	.177	0.09
	Housing: Females[a]	$F_{(1, 19)} = 7.91$	.011[#]	0.29
	Housing: Males[a]	$F_{(1, 19)} = 0.79$	.384	0.04
	Sex x ToD	$F_{(1, 19)} < 0.01$	.948	< 0.01
	Sex x Session	$F_{(3.438, 65.313)} = 1.16$	.334	0.06
	Housing x ToD	$F_{(1, 19)} = 0.04$	.851	< 0.01
	Housing x Session	$F_{(3.438, 65.313)} = 1.24$	.284	0.06
	ToD x Session	$F_{(3.438, 65.313)} = 1.92$	.127	0.09
	Sex x Housing x ToD	$F_{(1, 19)} = 0.36$	.557	0.02
	Sex x Housing x Session	$F_{(3.438, 65.313)} = 0.50$	.710	0.03
	Sex x ToD x Session	$F_{(3.438, 65.313)} = 0.76$	.538	0.04
	Housing x ToD x Session	$F_{(3.438, 65.313)} = 0.19$	.923	0.01
	Sex x Housing x ToD x Session	$F_{(3.438, 65.313)} = 0.98$	.418	0.05
Proportion of open-door time with nose in door	Sex	$F_{(1, 19)} = 1.30$	.269	0.06
	Housing	$F_{(1, 19)} = 2.63$	.121	0.12
	ToD	$F_{(1, 19)} = 1.84$	.190	0.09
	Session	$F_{(7, 133)} = 3.09$	.005*	0.14
	Session: Linear trend	$F_{(1, 19)} = 11.59$	.003*	0.38
	Session: Quadratic trend	$F_{(1, 19)} = 1.51$	.234	0.07
	Sex x Housing	$F_{(1, 19)} = 0.87$	.196	0.09
	Housing: Females[a]	$F_{(1, 19)} = 3.16$	.092	0.14
	Housing: Males[a]	$F_{(1, 19)} = 0.25$	.625	0.01
	Sex x ToD	$F_{(1, 19)} = 0.70$	.414	0.04
	Sex x Session	$F_{(7, 133)} = 1.18$	.317	0.06
	Housing x ToD	$F_{(1, 19)} = 0.16$	.697	0.01
	Housing x Session	$F_{(7, 133)} = 0.42$	.886	0.02
	ToD x Session	$F_{(7, 133)} = 0.73$	.643	0.04
	Sex x Housing x ToD	$F_{(1, 19)} = 1.79$	.196	0.09
	Sex x Housing x Session	$F_{(7, 133)} = 0.65$	.714	0.03
	Sex x ToD x Session	$F_{(7, 133)} = 0.44$	.876	0.02
	Housing x ToD x Session	$F_{(7, 133)} = 0.29$	.957	0.02
	Sex x Housing x ToD x Session	$F_{(7, 133)} = 1.26$	.275	0.06
Total time with nose in closed door	Sex	$F_{(1, 19)} = 6.50$	.020*	0.26
	Housing	$F_{(1, 19)} = 0.40$	.534	0.02
	ToD	$F_{(1, 19)} = 0.08$	.785	< 0.01
	Session	$F_{(7, 133)} = 0.72$	.659	0.04
	Sex x Housing	$F_{(1, 19)} = 0.58$	.457	0.03
	Housing: Females[a]	$F_{(1, 19)} = 0.01$	.931	< 0.01
	Housing: Males[a]	$F_{(1, 19)} = 1.01$	.328	0.05

	Sex x ToD	$F_{(1, 19)} = 0.09$	.769	0.01
	Sex x Session	$F_{(7, 133)} = 1.28$	.267	0.06
	Housing x ToD	$F_{(1, 19)} < 0.01$	.951	< 0.01
	Housing x Session	$F_{(7, 133)} = 0.79$	.593	0.04
	ToD x Session	$F_{(7, 133)} = 0.52$	.819	0.03
	Sex x Housing x ToD	$F_{(1, 19)} = 0.01$	.919	< 0.01
	Sex x Housing x Session	$F_{(7, 133)} = 0.52$	.820	0.03
	Sex x ToD x Session	$F_{(7, 133)} = 1.11$	.359	0.06
	Housing x ToD x Session	$F_{(7, 133)} = 0.54$	.800	0.03
	Sex x Housing x ToD x Session	$F_{(7, 133)} = 0.64$	.719	0.03
Proportion of closed-door time with nose in door	Sex	$F_{(1, 19)} < 0.01$	.992	< 0.01
	Housing	$F_{(1, 19)} = 0.74$	.400	0.04
	ToD	$F_{(1, 19)} = 0.13$	.727	0.01
	Session	$F_{(7, 133)} = 0.91$	.499	0.05
	Sex x Housing	$F_{(1, 19)} = 3.02$	.098	0.14
	Housing: Females[a]	$F_{(1, 19)} = 3.26$	.087	0.15
	Housing: Males[a]	$F_{(1, 19)} = 0.40$	.535	0.02
	Sex x ToD	$F_{(1, 19)} = 0.73$	.405	0.04
	Sex x Session	$F_{(7, 133)} = 0.75$	.630	0.04
	Housing x ToD	$F_{(1, 19)} = 0.01$	.943	< 0.01
	Housing x Session	$F_{(7, 133)} = 0.62$	.737	0.03
	ToD x Session	$F_{(7, 133)} = 1.47$	.182	0.07
	Sex x Housing x ToD	$F_{(1, 19)} = 0.01$	.943	< 0.01
	Sex x Housing x Session	$F_{(7, 133)} = 0.49$	.839	0.03
	Sex x ToD x Session	$F_{(7, 133)} = 0.85$	.552	0.04
	Housing x ToD x Session	$F_{(7, 133)} = 1.05$	.403	0.05
	Sex x Housing x ToD x Session	$F_{(7, 133)} = 1.27$	.272	0.06

2. Acquisition (Phase 1a) – ToD analyses and results

Figure S4 depicts the impacts of ToD and interactions with ToD during Phase 1a. Across acquisition sessions, rats tested during the light phase spent a greater proportion of open-door time with their noise in the door than rats tested in the dark phase (Figure S4G). The reduction in locomotion across consecutive sessions was more marked for rats tested in the dark phase than those tested in the light phase (Figure S4D). A significant *Sex* x *ToD* x *Time* interaction was found (Figure S4H), indicating that during the light phase, female rats spent a markedly greater portion of open-door time with their nose in open door than male rats during the first three compared to the last three social rewards obtained. The proportion of closed door time rats spent with their nose in the closed door varied significant over sessions, and this pattern differed by both sex and ToD (Figure S4J). This indicates that the increase in the difference between male and females towards the later acquisition sessions was greater for rats tested in the light phase compared to the dark phase. However, no significant main effect of ToD or other interactions with ToD were found.

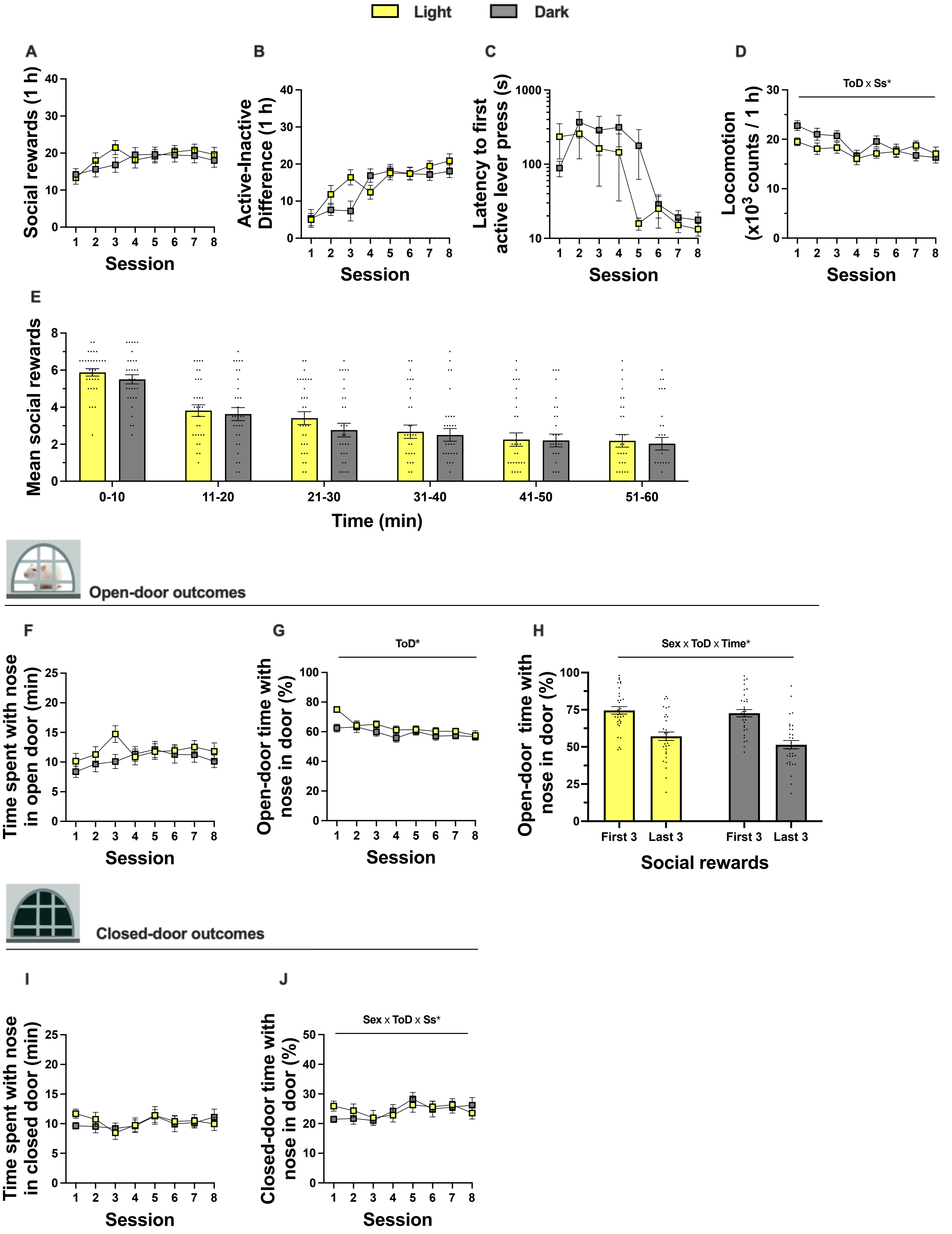

Light
Dark
A
Social rewards (1 h)
Session
B
Active-Inactive Difference (1 h)
Session
C
Latency to first active lever press (s)
Session
D
ToD x Ss*
Locomotion ($x10^3$ counts / 1 h)
Session
E
Mean social rewards
0-10
11-20
21-30
31-40
41-50
51-60
Time (min)
Open-door outcomes
F
Time spent with nose in open door (min)
Session
G
ToD*
Open-door time with nose in door (%)
Session
H
Sex x ToD x Time*
Open-door time with nose in door (%)
First 3
Last 3
First 3
Last 3
Social rewards
Closed-door outcomes
I
Time spent with nose in closed door (min)
Session
J
Sex x ToD x Ss*
Closed-door time with nose in door (%)
Session

Figure S4. The influence of time-of-day on outcomes of acquisition in social operant conditioning. Effects of the experimental parameter time-of-day on social rewards (A), difference in active-inactive lever presses (B), latency to first active lever press (C), locomotor activity (D), within-session time course mean social rewards (E), total time with nose in open door (F), proportion of open door time with nose in open door (G), within-session proportion of open door time with nose in open door (H), total time with nose in closed door (I), and proportion of closed door time with nose in closed door (J). Sample sizes were $n = 8$ per factorial condition, except for locomotion, total time with nose in open door, total time with nose in closed door, and proportion of closed door time with nose in closed door (F-Iso-L: $n = 7$, F-Pair-L: $n = 7$, F-Iso-D: $n = 6$, F-Pair-D: $n = 6$, M-Iso-L: $n = 7$, M-Pair-L: $n = 8$, M-Iso-D: $n = 6$, M-Pair-D: $n = 6$); and proportion of open door time with nose in open door (F-Iso-L: $n = 7$, F-Pair-L: $n = 7$, F-Iso-D: $n = 6$, F-Pair-D: $n = 6$, M-Iso-L: $n = 6$, M-Pair-L: $n = 8$, M-Iso-D: $n = 6$, M-Pair-D: $n = 4$). Data represent mean values ± S.E.M. and individual data points represent individual subject data. Statistical significance is indicated by the following: Sex – biological sex; ToD – time-of-day; Ss – session, Time – timepoint during session. Level of statistical significance is indicated by the number of * symbols: one – $p < .05$. Note that only ToD main and interaction effects with ToD are depicted; all other effects are illustrated in Figure 2.

3. ToD Switch (Phase 1b) – ToD switch analyses and results

Given the absence of clear ToD effects on social operant outcomes in Phase 1a, Phase 1b aimed to determine whether the development of circadian entrainment of social operant responding during acquisition may explain this absence of ToD effects. Figure S5 illustrates the results from the ToD switch conducted during Phase 1b and Table S2 summarises the results of corresponding statistical analyses.

No main effect of ToD was found for any outcomes, however switching the ToD of testing did impact locomotor activity: rats demonstrated elevated locomotion post-switch compared to pre-switch (Figure S5G and H). Further, the effect of switching ToD depended on the ToD rats were previously tested on; rats that experienced delayed testing post-switch (i.e., pre-switch light phase) obtained fewer social rewards and a lower active-inactive difference, whereas rats that experienced advanced testing post-switch (i.e., pre-switch dark phase) achieved more social rewards and a greater active-inactive difference (Figure S5A-D). The impact of switching ToD depended on sex; male rats obtained more social rewards post-switch, whereas female rats obtained fewer social rewards. Additionally, a significant *Housing* x *ToD* x *Switch* interaction effect was observed; the pattern of lower time spent with nose in open door for rats previously tested under the light phase and higher time spent for rats previously tested under the dark phase was more marked for isolated rats than for pair-housed rats (Figure S5I and J).

Averaged over pre- and post-switch sessions, a sex difference was found; relative to male rats, females obtained more social rewards, demonstrated a greater active-inactive difference in lever presses and shorter latency to first press, exhibited higher locomotor activity, spent greater total time with their nose in open door, and spent less total time with their nose in the closed door. Across both sessions, an effect of housing condition was also observed; isolated rats obtained more social rewards, exhibited greater active-inactive

difference in lever presses and higher locomotor activity, and spent more total time and a greater proportion of open-door time with noses in the open door. For several outcomes, the influence of housing condition interacted with biological sex. The isolation-induced increase in total time with nose in open door and proportion of open-door time with nose in open door was greater for female rats compared to males. Further, the isolation-induced increase in social rewards, locomotion, total time with nose in open door, and proportion of open-door time with nose in open door was only found for female rats and not males.

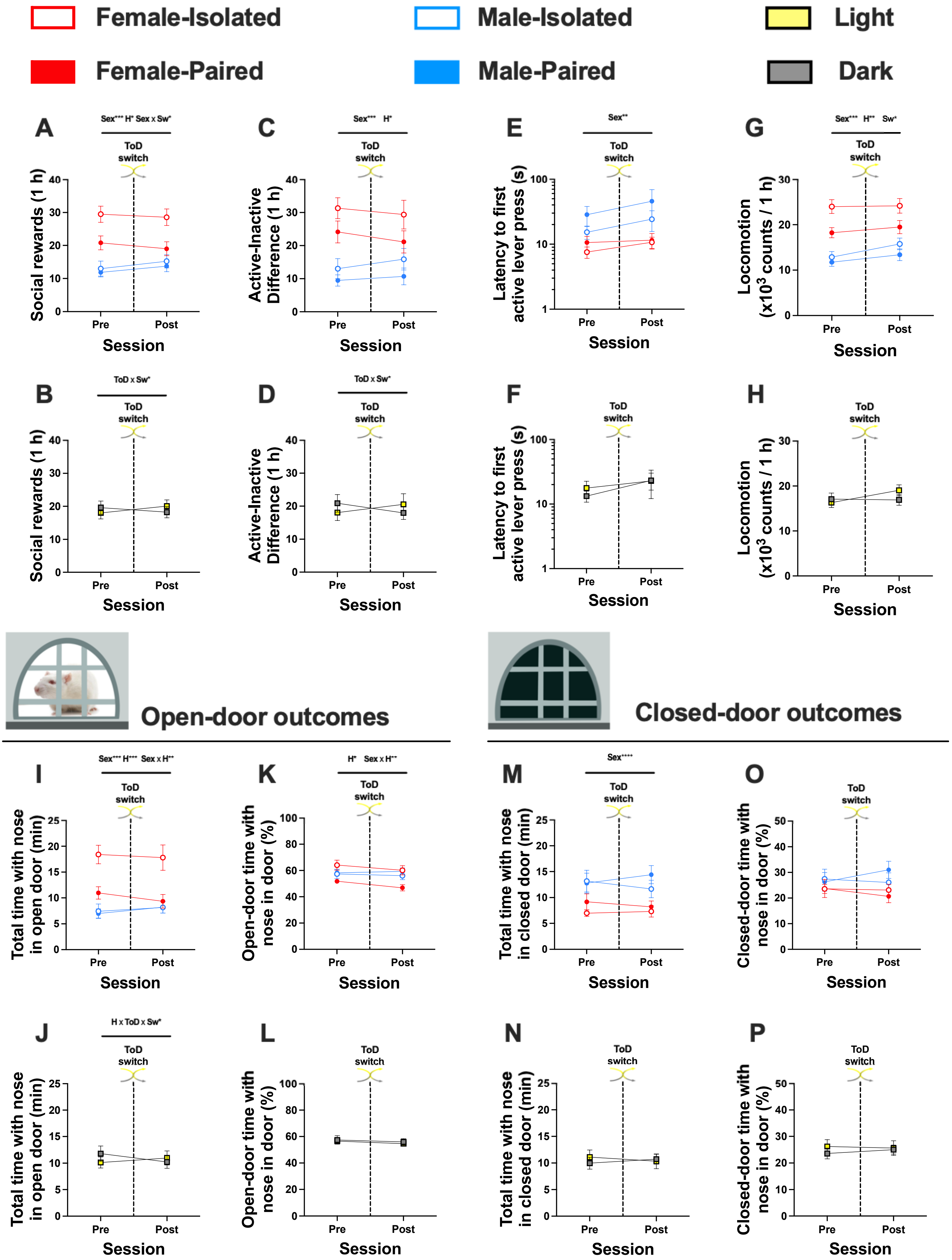
Female-Isolated
Male-Isolated
Light
Female-Paired
Male-Paired
Dark
A
Sex*** H* Sex x Sw*
ToD switch
Social rewards (1 h)
Session
Pre
Post
C
Sex*** H*
Active-Inactive Difference (1 h)
E
Sex**
Latency to first active lever press (s)
G
Sex*** H** Sw*
Locomotion (x10³ counts / 1 h)
B
ToD x Sw*
D
ToD x Sw*
F
H
Open-door outcomes
Closed-door outcomes
I
Sex*** H*** Sex x H**
Total time with nose in open door (min)
K
H* Sex x H**
Open-door time with nose in door (%)
M
Sex****
Total time with nose in closed door (min)
O
Closed-door time with nose in door (%)
J
H x ToD x Sw*
L
N
P

Figure S5. The influence of switching time-of-day on outcomes in social operant conditioning. Effects of switching time-of-day, in addition to experimental parameters of biological sex, housing conditions, and time-of-day, on social rewards (A and B), difference in active-inactive lever presses (C and D), latency to first active lever press (E and F), locomotor activity (G and H), total time with nose in open door (I and J), proportion of open door time with nose in open door (K and L), total time with nose in closed door (M and N), and proportion of closed door time with nose in closed door (O and P). Sample sizes were n = 8 per factorial condition, except for locomotion, total time with nose in open door, proportion of open door time with nose in open door, total time with nose in closed door, and proportion of closed door time with nose in closed door (F-Iso-L: n = 7, F-Pair-L: n = 7, F-Iso-D: n = 6, F-Pair-D: n = 8, M-Iso-L: n = 8, M-Pair-L: n = 8, M-Iso-D: n = 7, M-Pair-D: n = 8). Data represent mean values ± S.E.M and statistical significance is indicated by the following: Sex – biological sex; H – housing condition; ToD – time-of-day; Sw – ToD switch. Level of statistical significance is indicated by the number of * symbols: one – $p < .05$, two – $p < .01$, three – $p < .001$. Note that only ToD main and interaction effects are depicted above ToD graphs; all other effects are illustrated above the Sex x Housing graphs.

Table S2. Summary of statistical analyses for Phase 1b (ToD switch).

Outcome	Effect	F statistic	p-value	Partial η^2
Time-of-Day switch (Phase 1b)				
**Social rewards**	Sex	$F_{(1, 56)} = 30.03$	< .001*	0.35
	Housing	$F_{(1, 56)} = 6.76$	.012*	0.11
	ToD	$F_{(1, 56)} < 0.01$	.951	< 0.01
	Switch	$\mathrm{F}_{(1, 56)} = 0.22$	.639	< 0.01
	Sex x Housing	$F_{(1, 56)} = 3.79$	.057	0.06
	Housing: Females[a]	$F_{(1, 56)} = 10.33$	.002[#]	0.16
	Housing: Males[a]	$F_{(1, 56)} = 0.21$	.646	< 0.01
	Sex x ToD	$F_{(1, 56)} = 0.43$	.516	0.01
	Sex x Switch	$\mathrm{F}_{(1, 56)} = 5.56$	.022*	0.09
	Housing x ToD	$F_{(1, 56)} = 0.02$	.889	< 0.01
	Housing x Switch	$\mathrm{F}_{(1, 56)} = 0.18$	.670	< 0.01
	ToD x Switch	$\mathrm{F}_{(1, 56)} = 5.56$	.022*	0.09
	Sex x Housing x ToD	$F_{(1, 56)} = 0.26$	.609	0.01
	Sex x Housing x Switch	$\mathrm{F}_{(1, 56)} = 0.03$	.864	< 0.01
	Sex x ToD x Switch	$\mathrm{F}_{(1, 56)} = 2.00$	.163	0.04
	Housing x ToD x Switch	$\mathrm{F}_{(1, 56)} = 2.38$	.128	0.04
	Sex x Housing x ToD x Switch	$\mathrm{F}_{(1, 56)} = 1.66$	.204	0.03
**Difference in active-inactive lever presses**	Sex	$F_{(1, 56)} = 23.57$	< .001*	0.30
	Housing	$F_{(1, 56)} = 4.25$	.044*	0.07
	ToD	$F_{(1, 56)} < 0.01$	.970	< 0.01

	Switch	$F_{(1, 56)} = 0.02$	.878	< 0.01
	Sex x Housing	$F_{(1, 56)} = 0.33$	.571	0.01
	Housing: Females[a]	$F_{(1, 56)} = 3.47$	.068	0.06
	Housing: Males[a]	$F_{(1, 56)} = 1.11$	.296	0.02
	Sex x ToD	$F_{(1, 56)} = 0.47$	.495	0.01
	Sex x Switch	$F_{(1, 56)} = 2.97$	.090	0.05
	Housing x ToD	$F_{(1, 56)} = 0.01$	.920	< 0.01
	Housing x Switch	$F_{(1, 56)} = 0.26$	.611	0.01
	ToD x Switch	$F_{(1, 56)} = 4.23$	.044*	0.07
	Sex x Housing x ToD	$F_{(1, 56)} = 0.63$	.431	0.01
	Sex x Housing x Switch	$F_{(1, 56)} = 0.01$	.915	< 0.01
	Sex x ToD x Switch	$F_{(1, 56)} = 1.80$	.185	0.03
	Housing x ToD x Switch	$F_{(1, 56)} = 0.05$	.822	< 0.01
	Sex x Housing x ToD x Switch	$F_{(1, 56)} = 3.22$	.078	0.05
**Latency to active lever first press *(log transform)***	Sex	$F_{(1, 56)} = 10.16$	.002*	0.15
	Housing	$F_{(1, 56)} = 0.48$	.493	0.01
	ToD	$F_{(1, 56)} = 0.12$	.734	< 0.01
	Switch	$F_{(1, 56)} = 0.33$	.569	0.01
	Sex x Housing	$F_{(1, 56)} = 0.03$	.870	< 0.01
	Housing: Females[a]	$F_{(1, 56)} = 0.14$	.712	< 0.01
	Housing: Males[a]	$F_{(1, 56)} = 0.37$	.548	0.01
	Sex x ToD	$F_{(1, 56)} = 0.36$	.552	0.01
	Sex x Switch	$F_{(1, 56)} = 0.47$	.497	0.01
	Housing x ToD	$F_{(1, 56)} = 0.17$	.679	< 0.01
	Housing x Switch	$F_{(1, 56)} = 1.40$	.243	0.02
	ToD x Switch	$F_{(1, 56)} = 2.25$	.139	0.04
	Sex x Housing x ToD	$F_{(1, 56)} = 1.97$	.166	0.03
	Sex x Housing x Switch	$F_{(1, 56)} = 0.01$	.923	< 0.01
	Sex x ToD x Switch	$F_{(1, 56)} = 0.23$	.632	< 0.01
	Housing x ToD x Switch	$F_{(1, 56)} = 0.11$	.737	< 0.01
	Sex x Housing x ToD x Switch	$F_{(1, 56)} = 3.39$	.071	0.06
**Locomotion**	Sex	$F_{(1, 51)} = 45.43$	< .001*	0.47
	Housing	$F_{(1, 51)} = 8.71$	.005*	0.15
	ToD	$F_{(1, 51)} = 0.48$	.490	0.01
	Switch	$F_{(1, 51)} = 5.21$	.027*	0.09
	Sex x Housing	$F_{(1, 51)} = 1.78$	.188	0.03
	Housing: Females[a]	$F_{(1, 51)} = 8.71$	.005[#]	0.15
	Housing: Males[a]	$F_{(1, 51)} = 1.39$	.245	0.03
	Sex x ToD	$F_{(1, 51)} = 0.09$	.768	< 0.01
	Sex x Switch	$F_{(1, 51)} = 1.10$	.299	0.02
	Housing x ToD	$F_{(1, 51)} = 0.02$	.897	< 0.01

	Housing x Switch	$F_{(1, 51)} = 0.04$	.848	< 0.01
	ToD x Switch	$F_{(1, 51)} = 3.55$	.065	0.07
	Sex x Housing x ToD	$F_{(1, 51)} = 0.05$	.821	< 0.01
	Sex x Housing x Switch	$F_{(1, 51)} = 0.95$	.334	0.02
	Sex x ToD x Switch	$F_{(1, 51)} = 2.06$	.158	0.04
	Housing x ToD x Switch	$F_{(1, 51)} = 0.31$	.580	0.01
	Sex x Housing x ToD x Switch	$F_{(1, 51)} = 1.28$	.263	0.03
**Total time with nose in open door**	Sex	$F_{(1, 51)} = 22.89$	< .001*	0.31
	Housing	$F_{(1, 51)} = 8.45$	< .001*	0.14
	ToD	$F_{(1, 51)} = 0.01$	.944	< 0.01
	Switch	$F_{(1, 51)} = 0.04$	.842	< 0.01
	Sex x Housing	$F_{(1, 51)} = 7.43$	.009*	0.13
	Housing: Females[a]	$F_{(1, 51)} = 15.04$	< .001[#]	0.23
	Housing: Males[a]	$F_{(1, 51)} = 0.02$	.896	< 0.01
	Sex x ToD	$F_{(1, 51)} = 0.56$	.459	0.01
	Sex x Switch	$F_{(1, 51)} = 1.87$	.177	0.04
	Housing x ToD	$F_{(1, 51)} = 0.13$	.717	< 0.01
	Housing x Switch	$F_{(1, 51)} = 0.02$	.878	< 0.01
	ToD x Switch	$F_{(1, 51)} = 2.84$	.098	0.05
	Sex x Housing x ToD	$F_{(1, 51)} = 1.15$	.289	0.02
	Sex x Housing x Switch	$F_{(1, 51)} = 0.51$	.479	0.01
	Sex x ToD x Switch	$F_{(1, 51)} = 1.21$	.277	0.02
	Housing x ToD x Switch	$F_{(1, 51)} = 4.33$	.042*	0.08
	Sex x Housing x ToD x Switch	$F_{(1, 51)} = 0.01$	.938	< 0.01
**Proportion of open-door time with nose in door**	Sex	$F_{(1, 51)} = 0.35$	.556	0.01
	Housing	$F_{(1, 51)} = 4.34$	.042*	0.08
	ToD	$F_{(1, 51)} = 0.78$	.381	0.02
	Switch	$F_{(1, 51)} = 0.83$	.367	0.02
	Sex x Housing	$F_{(1, 51)} = 9.04$	.004*	0.15
	Housing: Females[a]	$F_{(1, 51)} = 12.28$	.001[#]	0.19
	Housing: Males[a]	$F_{(1, 51)} = 0.45$	.505	0.01
	Sex x ToD	$F_{(1, 51)} = 0.13$	.723	< 0.01
	Sex x Switch	$F_{(1, 51)} = 1.22$	.274	0.02
	Housing x ToD	$F_{(1, 51)} = 0.04$	.847	< 0.01
	Housing x Switch	$F_{(1, 51)} = 0.08$	.777	< 0.01
	ToD x Switch	$F_{(1, 51)} = 0.20$	.659	< 0.01
	Sex x Housing x ToD	$F_{(1, 51)} = 0.81$	.372	0.02
	Sex x Housing x Switch	$F_{(1, 51)} = 0.16$	.690	< 0.01
	Sex x ToD x Switch	$F_{(1, 51)} = 0.13$	.721	< 0.01
	Housing x ToD x Switch	$F_{(1, 51)} = 2.39$	.128	0.05
	Sex x Housing x ToD x Switch	$F_{(1, 51)} = 1.29$	.262	0.03

**Total time with nose in closed door**	Sex	$F_{(1, 51)} = 17.39$	< .001*	0.25
	Housing	$F_{(1, 51)} = 1.24$	.272	0.02
	ToD	$F_{(1, 51)} = 0.07$	.787	< 0.01
	Switch	$F_{(1, 51)} < 0.01$	.950	< 0.01
	Sex x Housing	$F_{(1, 51)} = 0.05$	.823	< 0.01
	Housing: Females[a]	$F_{(1, 51)} = 0.37$	.544	0.01
	Housing: Males[a]	$F_{(1, 51)} = 0.94$	.336	0.02
	Sex x ToD	$F_{(1, 51)} = 0.08$	.783	< 0.01
	Sex x Switch	$F_{(1, 51)} = 0.32$	.574	0.01
	Housing x ToD	$F_{(1, 51)} = 0.98$	.326	0.02
	Housing x Switch	$F_{(1, 51)} < 0.01$	.981	< 0.01
	ToD x Switch	$F_{(1, 51)} = 0.10$	.752	< 0.01
	Sex x Housing x ToD	$F_{(1, 51)} < 0.01$	.948	< 0.01
	Sex x Housing x Switch	$F_{(1, 51)} = 1.23$	.272	0.02
	Sex x ToD x Switch	$F_{(1, 51)} = 0.36$	.549	0.01
	Housing x ToD x Switch	$F_{(1, 51)} = 0.36$	.551	0.01
	Sex x Housing x ToD x Switch	$F_{(1, 51)} < 0.01$	.963	< 0.01
**Proportion of closed-door time with nose in door**	Sex	$F_{(1, 51)} = 3.87$	.055	0.07
	Housing	$F_{(1, 51)} = 0.04$	.851	< 0.01
	ToD	$F_{(1, 51)} = 0.38$	.539	0.01
	Switch	$F_{(1, 51)} = 0.02$	.886	< 0.01
	Sex x Housing	$F_{(1, 51)} = 0.69$	.411	0.01
	Housing: Females[a]	$F_{(1, 51)} = 0.20$	.661	< 0.01
	Housing: Males[a]	$F_{(1, 51)} = 0.55$	.462	0.01
	Sex x ToD	$F_{(1, 51)} = 0.20$	.654	0.01
	Sex x Switch	$F_{(1, 51)} = 1.23$	.273	0.02
	Housing x ToD	$F_{(1, 51)} = 0.39$	.534	0.01
	Housing x Switch	$F_{(1, 51)} < 0.01$	.950	< 0.01
	ToD x Switch	$F_{(1, 51)} = 0.01$	.926	< 0.01
	Sex x Housing x ToD	$F_{(1, 51)} = 0.17$	.682	< 0.01
	Sex x Housing x Switch	$F_{(1, 51)} = 1.43$	.238	0.03
	Sex x ToD x Switch	$F_{(1, 51)} = 0.20$	.655	< 0.01
	Housing x ToD x Switch	$F_{(1, 51)} = 1.26$	.267	0.02
	Sex x Housing x ToD x Switch	$F_{(1, 51)} < 0.01$	.999	< 0.01

[a]Simple effects contrast
[#]Statistically significant with Bonferroni correction applied to simple effects contrasts ($p < .025$)

4. Time-of-day, circadian phase, and entrainment: Discussion and implications

To the authors' best knowledge, the current study provides the first systematic investigation into the effect of ToD and interactions of ToD with biological sex on outcomes in the social operant conditioning model. Based on ethological crepuscularity in rodent circadian rhythms, testing during the early-dark phase has been proposed as an optimal ToD to conduct behavioural testing (1). In contrast, the current findings demonstrate little to no differences across most social operant parameters between light and dark phase testing. Rather, the results suggest that due to circadian entrainment of social operant responding (i.e., that subjects learn when during the light cycle to expect social operant testing), ToD is not a critical factor that influences social motivation, provided that the ToD of testing is maintained consistently across consecutive sessions. However, as rats in the current study only experienced brief 1-h social operant sessions, this interpretation of a lack of ToD effects on social motivation is limited. Future research should offer subjects multiple consecutive 24-h periods within social operant chambers or develop and utilise an automated social operant home cage-coupled system (2) under standard light cycling conditions to provide a more nuanced assessment of whether ToD influences social motivation, and whether sex differences exist in the circadian patterns observed.

This finding of socially-derived circadian entrainment aligns with research in animals and humans on social zeitgebers (i.e., time-cues) wherein social stimuli are proposed to serve as cues that entrain behaviour to the local environmental context (3-6). A common example of this phenomenon is a co-sleeping individual's early morning awakening entraining the wake time and activity of their bedpartner. Although light exposure is considered the prevailing zeitgeber that entrains circadian patterns of activity, for many mammalian species including humans, the activity of other conspecifics with which they interact can alter this photic-dominant circadian control of activity (3, 4). Social zeitgeber theory (6) postulates that

disruptions to individuals' social routines—and consequently biological circadian rhythms—contribute to the development of dysregulated affect in unipolar and bipolar mood disorders (5). Experimental manipulations of time-of-day for subjects within the social operant conditioning model may offer a preclinical avenue for research into links between socially-induced circadian disruption and mood disorders.

5. Supplementary correlational analyses of social operant data

5.1. Relationships between behavioural economics outcomes from between- and with-session procedures (Phases 2 and 3).

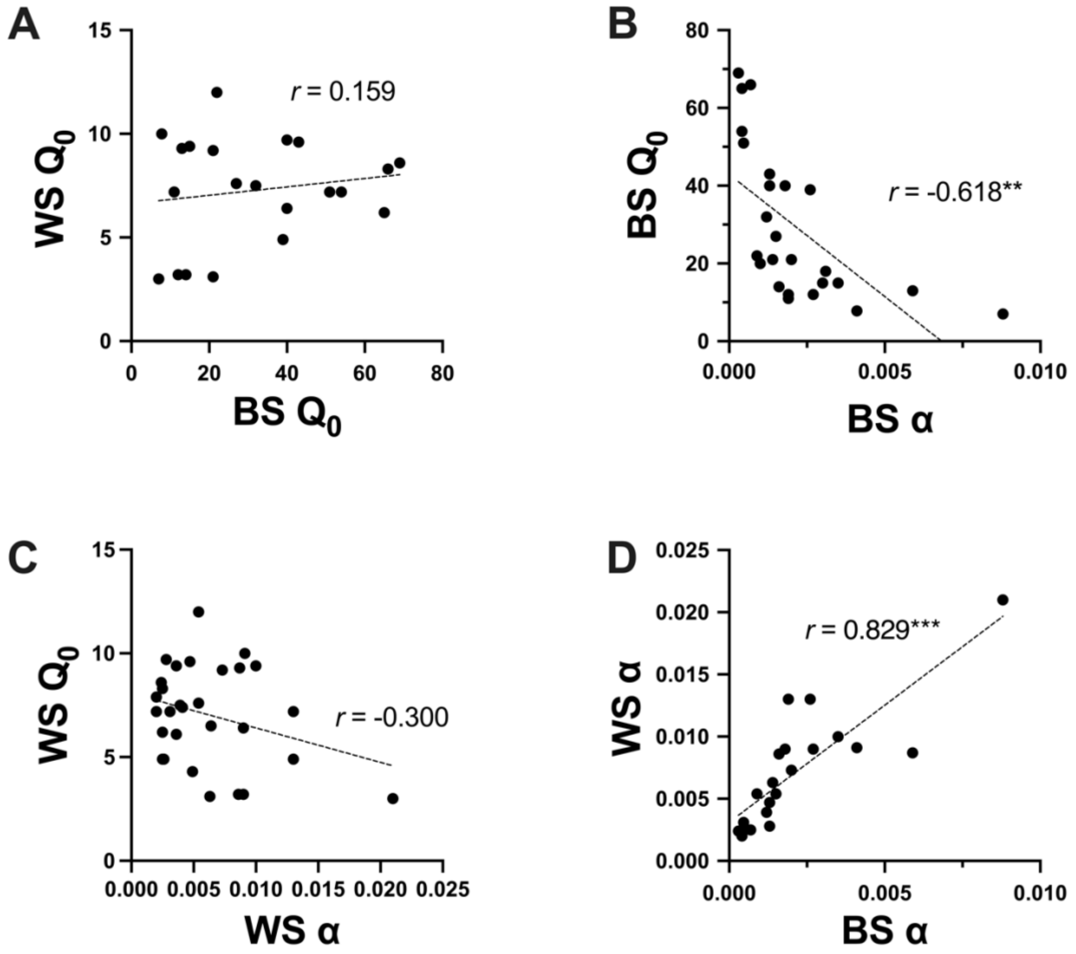

Figure S6. Scatterplots of intra-subject correlations between behavioural economics outcomes from between- and within-session procedures. (A) Pearson's correlation of demand at null cost (Q_0) values for between- and within-session procedures (*n* = 21); (B) Pearson's correlation of demand at null cost (Q_0) and demand elasticity (α) for the between-session procedure (*n* = 25); (C) Pearson's correlation of demand at null cost (Q_0) and demand elasticity (α) for the within-session procedure (*n* = 29); and (D) Pearson's correlation of demand elasticity (α) values for between- and within-session procedures (*n* = 21). Each data point represents intra-subject data for both outcomes; different *n* are due to exclusion of subject data from demand curves with R^2 values < 0.6. Level of statistical significance is indicated by the number of * symbols: one – $p < .05$, two – $p < .01$, three – $p < .001$.

5.2. Predictive relationships between social operant outcomes from Phase 1 and behavioural economics outcomes from Phases 2 and 3.

Given that acquisition of social operant responding must be conducted prior to commencing behavioural economics approaches (between- or within-session), and that these processes depend on inclusion of highly socially motivated subjects, social outcome data from acquisition can be used to predict which subjects will perform better during behavioural economics. As such, correlational analyses of subjects included in all phases of the experiment were conducted to identify potential predictive outcomes and the timepoints during acquisition by which they are relatively stable in this predictive role. Note however, that as the following analyses only include subjects from Phases 2 and 3 (i.e., the top 50% of responders), these predictions may not generalise to rats with low social motivation.

For between-session social operant behavioural economics, the best predictors of demand at null cost and demand elasticity were social rewards and locomotion, demonstrating positive and negative associations—respectively—that indicate that increased social rewards and locomotor activity predict increased motivation for social interaction (Table S3). While locomotion appears to begin a strong predictive value from Session 2 onwards, the predictive value of social rewards only appears to demonstrate some predictive stability around Session 4 of acquisition. No other social outcomes from acquisition appear to exhibit useful predictive value for between-session behavioural economic outcomes.

For within-session social operant behavioural economics, regardless of same- or opposite-sex stimuli, no social outcomes during acquisition effectively predicted demand at null cost, likely as an artefact of within-session experimental procedures (as discussed in the main text discussion). However, social rewards, locomotion, total time spent with nose in open door, and active-inactive difference all provide strong predictive value for demand elasticity from approximately acquisition session 2-3 onwards (Table S4). These negative

associations suggest that increases in social rewards/locomotion/time spent with nose in open door and active-inactive differences significantly predict lower demand elasticity (i.e., less sensitivity to increases in cost and thus, higher social motivation).

Table S3. Correlation matrix of social operant outcomes from acquisition (Phase 1a) with between-session (Phase 2) behavioural economics outcomes.

Social operant outcome	Phase 1a Session	Demand at null cost (Q_0)		Demand elasticity (α)	
		Pearson's *r*	*p*	Pearson's *r*	*p*
Social rewards	*S1*	0.136	0.517	-0.285	0.167
	S2	0.303	0.141	-0.456	0.022
	S3	0.111	0.597	-0.334	0.103
	S4	0.482	0.015	-0.430	0.032
	S5	0.393	0.052	-0.408	0.043
	S6	0.560	0.004	-0.591	0.002
	S7	0.389	0.055	-0.298	0.148
	S8	0.484	0.014	-0.252	0.224
Locomotion	*S1*	0.460	0.021	-0.324	0.114
	S2	0.533	0.006	-0.434	0.030
	S3	0.518	0.008	-0.487	0.014
	S4	0.603	0.001	-0.476	0.016
	S5	0.469	0.018	-0.484	0.014
	S6	0.584	0.002	-0.460	0.021
	S7	0.522	0.007	-0.467	0.019
	S8	0.574	0.003	-0.393	0.052
%Open-door nose in door time	*S1*	-0.191	0.362	0.083	0.693
	S2	-0.065	0.756	-0.017	0.936
	S3	0.014	0.948	-0.158	0.450
	S4	-0.225	0.280	0.404	0.045
	S5	0.139	0.509	0.004	0.984
	S6	0.146	0.487	-0.027	0.897
	S7	0.028	0.894	0.224	0.282
	S8	-0.324	0.114	0.443	0.027
%Closed-door nose in door time	*S1*	-0.108	0.608	-0.123	0.559
	S2	-0.027	0.897	0.456	0.022
	S3	0.268	0.195	-0.390	0.054
	S4	-0.038	0.858	0.117	0.577
	S5	0.026	0.903	-0.136	0.518
	S6	0.121	0.563	-0.239	0.250
	S7	0.384	0.058	-0.255	0.219
	S8	0.186	0.373	-0.252	0.225
Total time nose in open door	*S1*	0.063	0.766	-0.249	0.230
	S2	0.240	0.249	-0.416	0.039
	S3	0.093	0.660	-0.327	0.111
	S4	0.432	0.031	-0.300	0.146
	S5	0.399	0.048	-0.379	0.061
	S6	0.744	< 0.001	-0.642	0.001
	S7	0.400	0.047	-0.178	0.395
	S8	0.352	0.084	-0.053	0.802
Total time nose in closed door	*S1*	-0.167	0.425	0.054	0.797
	S2	-0.152	0.467	0.580	0.002
	S3	0.147	0.483	-0.176	0.400
	S4	-0.204	0.327	0.243	0.241
	S5	-0.206	0.324	0.090	0.670
	S6	0.032	0.878	-0.046	0.826
	S7	0.071	0.734	-0.065	0.759
	S8	-0.144	0.491	-0.050	0.811
Latency to first active press	*S1*	-0.224	0.282	0.214	0.303
	S2	0.118	0.575	0.047	0.823
	S3	-0.060	0.776	-0.014	0.947
	S4	-0.096	0.649	-0.073	0.728
	S5	0.092	0.661	0.118	0.575
	S6	-0.036	0.863	0.197	0.344
	S7	-0.170	0.417	0.068	0.746
	S8	-0.279	0.176	0.308	0.134
Active-Inactive Difference	*S1*	-0.196	0.348	0.117	0.577
	S2	0.310	0.132	-0.276	0.182
	S3	0.175	0.403	-0.272	0.188
	S4	0.382	0.059	-0.320	0.119
	S5	0.231	0.266	-0.269	0.193
	S6	0.384	0.058	-0.375	0.065
	S7	0.314	0.126	-0.304	0.139
	S8	0.342	0.094	-0.188	0.367
Within-session Last 3-First 3 social reward %open door time with nose in door *(Days 7 and 8)*		-0.415	0.039	0.224	0.281

Table S4. Correlation matrix of social operant outcomes from Acquisition (Phase 1a) with within-session (Phase 3) behavioural economics outcomes.

Social operant outcome	Phase 1a Session	Same-sex stimulus				Opposite-sex stimulus			
		Demand at null cost (Q_0)		Demand elasticity (α)		Demand at null cost (Q_0)		Demand elasticity (α)	
		Pearson's *r*	*p*	Pearson's *r*	*p*	Pearson's *r*	*p*	Pearson's *r*	*p*
Social rewards	*S1*	0.121	0.532	-0.316	0.095	0.040	0.836	-0.216	0.236
	S2	0.123	0.526	-0.422	0.023	-0.084	0.664	-0.453	0.009
	S3	0.153	0.428	-0.410	0.027	-0.036	0.855	-0.552	0.001
	S4	0.235	0.220	-0.556	0.002	-0.043	0.823	-0.619	< 0.001
	S5	0.233	0.223	-0.611	0.000	-0.045	0.817	-0.650	< 0.001
	S6	0.173	0.370	-0.556	0.002	-0.028	0.885	-0.633	< 0.001
	S7	0.310	0.102	-0.602	0.001	-0.086	0.657	-0.661	< 0.001
	S8	0.363	0.053	-0.638	< 0.001	-0.061	0.752	-0.660	< 0.001
Locomotion	*S1*	0.160	0.406	-0.498	0.006	-0.033	0.864	-0.408	0.020
	S2	-0.105	0.587	-0.329	0.081	-0.204	0.288	-0.290	0.107
	S3	0.009	0.965	-0.457	0.013	-0.084	0.665	-0.482	0.005
	S4	0.203	0.290	-0.556	0.002	-0.120	0.537	-0.577	0.001
	S5	0.167	0.388	-0.555	0.002	0.055	0.776	-0.558	0.001
	S6	0.044	0.820	-0.317	0.094	-0.236	0.218	-0.408	0.021
	S7	0.167	0.388	-0.456	0.013	0.001	0.995	-0.526	0.002
	S8	0.222	0.247	-0.579	0.001	-0.089	0.645	-0.602	< 0.001
%Open-door nose in door time	*S1*	0.056	0.774	-0.021	0.914	0.145	0.454	-0.181	0.321
	S2	-0.121	0.531	0.212	0.270	-0.064	0.743	0.272	0.132
	S3	-0.053	0.784	0.070	0.718	-0.034	0.859	0.221	0.224
	S4	0.026	0.894	0.044	0.820	0.116	0.548	0.182	0.320
	S5	0.095	0.622	-0.066	0.733	0.268	0.160	-0.065	0.724
	S6	0.092	0.634	-0.083	0.669	-0.026	0.894	0.105	0.568
	S7	-0.039	0.841	0.154	0.425	0.039	0.840	0.023	0.902
	S8	-0.131	0.500	0.301	0.113	-0.075	0.699	0.377	0.033
%Closed-door nose in door time	*S1*	-0.017	0.929	-0.204	0.289	0.173	0.369	-0.276	0.126
	S2	0.133	0.491	-0.268	0.160	-0.010	0.960	-0.084	0.648
	S3	0.112	0.564	-0.147	0.448	0.187	0.331	-0.150	0.413
	S4	0.014	0.944	-0.236	0.217	0.017	0.932	-0.128	0.485
	S5	0.085	0.660	-0.222	0.247	0.284	0.135	-0.055	0.766
	S6	-0.303	0.111	0.273	0.152	0.015	0.940	0.407	0.021
	S7	-0.227	0.236	0.139	0.474	0.192	0.319	0.117	0.523
	S8	-0.180	0.349	0.047	0.809	0.271	0.155	0.160	0.383
Total time nose in open door	*S1*	0.152	0.430	-0.330	0.081	0.076	0.697	-0.288	0.110
	S2	0.080	0.681	-0.354	0.060	-0.048	0.803	-0.353	0.048
	S3	0.112	0.564	-0.366	0.051	-0.065	0.739	-0.444	0.011
	S4	0.218	0.256	-0.551	0.002	0.008	0.966	-0.574	0.001
	S5	0.192	0.319	-0.586	0.001	0.033	0.865	-0.587	< 0.001
	S6	0.265	0.165	-0.538	0.003	-0.035	0.855	-0.537	0.002
	S7	0.253	0.185	-0.464	0.011	-0.057	0.769	-0.556	0.001
	S8	0.338	0.073	-0.556	0.002	-0.051	0.792	-0.562	0.001
Total time nose in closed door	*S1*	-0.152	0.431	0.046	0.812	0.140	0.469	-0.097	0.596
	S2	0.098	0.615	-0.035	0.859	0.012	0.950	0.125	0.494
	S3	0.050	0.798	0.120	0.534	0.208	0.278	0.181	0.321
	S4	-0.030	0.875	0.042	0.827	0.052	0.787	0.190	0.299
	S5	-0.013	0.946	0.117	0.546	0.283	0.137	0.252	0.164
	S6	-0.060	0.759	0.449	0.015	0.055	0.776	0.516	0.002
	S7	-0.351	0.062	0.479	0.009	0.204	0.289	0.436	0.013
	S8	-0.321	0.090	0.392	0.035	0.241	0.208	0.434	0.013
Latency to first active press	*S1*	0.004	0.984	0.139	0.472	-0.190	0.325	0.003	0.989
	S2	0.270	0.156	0.003	0.987	0.101	0.601	-0.081	0.658
	S3	-0.416	0.025	0.711	< 0.001	-0.292	0.125	0.735	< 0.001
	S4	0.168	0.384	0.053	0.785	0.186	0.335	0.245	0.177
	S5	-0.067	0.731	0.072	0.712	0.110	0.571	0.024	0.898
	S6	0.130	0.502	0.219	0.253	-0.039	0.841	0.108	0.558
	S7	-0.173	0.370	0.367	0.050	0.266	0.163	0.292	0.104
	S8	0.119	0.540	0.506	0.005	0.411	0.027	0.499	0.004
Active-Inactive Difference	*S1*	0.161	0.403	-0.135	0.485	0.241	0.209	-0.090	0.626
	S2	0.068	0.726	-0.309	0.103	0.114	0.554	-0.350	0.050
	S3	0.155	0.421	-0.471	0.010	-0.061	0.753	-0.458	0.008
	S4	0.142	0.463	-0.501	0.006	0.006	0.977	-0.523	0.002
	S5	0.274	0.150	-0.597	0.001	-0.062	0.749	-0.573	0.001
	S6	0.109	0.574	-0.438	0.017	0.101	0.602	-0.501	0.003
	S7	0.290	0.128	-0.583	0.001	-0.087	0.654	-0.645	< 0.001
	S8	0.400	0.032	-0.608	< 0.001	-0.068	0.727	-0.622	< 0.001
Within-session Last 3-First 3 social reward %open door time with nose in door *(Days 7 and 8)*		-0.101	0.603	0.294	0.122	0.246	0.199	0.149	0.417

5.3. Relationships between behavioural economics outcomes for same- and opposite-sex stimuli from Phase 3.

Demand at null cost for same-sex stimuli was significantly positively associated with demand at null cost for opposite-sex stimuli, however it was not significantly associated with demand elasticity regardless of whether the social reward involved a same- or opposite-sex conspecific stimulus (Table S5). Similarly, demand elasticity for same-sex stimuli was significantly positively associated with demand elasticity for opposite-sex stimuli. Taken together, this provides further evidence that in the within-session social operant behavioural economics paradigm, Q_0 and α are mutually independent. The strength of the association in demand elasticity between same- and opposite-sex stimuli (r = 0.897), alongside the lack of effect of oestrus phase, could also indicate that the increased motivation for opposite-sex stimuli may not be due to conflation with sexual motivation, and rather may simply reflect elevated motivation for social novelty.

Table S5. Correlation matrix of behavioural economics outcomes from Phase 3.

P3 BE outcome	Stimulus sex	Demand at null cost (Q_0)				Demand elasticity (α)			
		Same-sex stimulus		*Opposite-sex stimulus*		*Same-sex stimulus*		*Opposite-sex stimulus*	
		Pearson's *r*	*p*	Pearson's *r*	*p*	Pearson's *r*	*p*	Pearson's *r*	*p*
Demand at null cost (Q_0)	*Same*								
	Opposite	0.423	0.022						
Demand elasticity (α)	*Same*	-0.300	0.114	-0.027	0.891				
	Opposite	-0.336	0.0747	-0.076	0.695	0.897	< 0.001		

5.4. Relationships between social rewards and video-derived open-door outcomes.

Across all experimental phases, the number of social rewards was strongly and positively associated with the total time spent with nose in open door. In contrast, the association between social rewards and the proportion of open-door time that rats spent with their nose in the door was weaker, more variable, and predominantly negative; that is, as the number of social rewards obtained increased, rats spent less of each open-door period in

interaction/investigation of the social stimulus (Table S6). This likely points toward an effect of social satiety within each session, as also indicated by the within-session first three-last three rewards analyses in Figure 2. This discrepancy highlights the complementary nature of using video recording in conjunction with social operant conditioning to provide a holistic analysis of social motivation within each session.

Table S6. Correlation matrix of social rewards with video-derived open-door outcomes from Phases 1, 2, and 3.

Social operant outcome	Phase 1a Session	Total time spent nose in open door		Proportion of open door time with nose in door	
		Pearson's *r*	*p*	Pearson's *r*	*p*
Social rewards	*S1*	0.925	< 0.001	-0.272	0.031
	S2	0.938	< 0.001	-0.292	0.023
	S3	0.894	< 0.001	-0.182	0.163
	S4	0.892	< 0.001	-0.146	0.257
	S5	0.917	< 0.001	-0.040	0.754
	S6	0.912	< 0.001	-0.177	0.193
	S7	0.907	< 0.001	0.072	0.570
	S8	0.922	< 0.001	0.034	0.792

Social operant outcome	Phase 2 FR	Total time spent nose in open door		Proportion of open door time with nose in door	
		Pearson's *r*	*p*	Pearson's *r*	*p*
Social rewards	*1*	0.898	< 0.001	-0.297	0.105
	2	0.915	< 0.001	-0.109	0.552
	4	0.836	< 0.001	-0.281	0.119
	6	0.829	< 0.001	-0.559	0.001
	9	0.931	< 0.001	-0.405	0.022
	12	0.934	< 0.001	-0.396	0.028

Social operant outcome	Phase 3	Total time spent nose in open door		Proportion of open door time with nose in door	
		Pearson's *r*	*p*	Pearson's *r*	*p*
Social rewards	*Same*	0.577	0.001	-0.435	0.013
	Opposite	0.586	0.001	-0.277	0.131

5.5. Relationships between behavioural economics outcomes and social rewards/video-derived open-door outcomes.

During between-session behavioural economics (Phase 2), demand at null cost and demand elasticity demonstrated moderate to strong positive and negative associations, respectively, with mean adjusted social rewards and total time spent with nose in open door at most FR schedules (Table S7). However, neither Q_0 nor α exhibited a stable relationship with the

proportion of open-door time that rats spent with their nose in the door across the FR schedules.

During within-session behavioural economics (Phase 3), demand elasticity exhibited strong negative associations with mean adjusted rewards and moderate negative associations with total time spent with nose in open door, for both same- and opposite-sex stimuli. This indicates that rats which are less sensitive to increases in cost, obtain more social rewards. Interestingly, a moderate positive association was found between α and the proportion of open-door time that rats spent with their nose in the door for same-sex but not opposite-sex stimuli. This may suggest that rats with higher social motivation (i.e., lower demand elasticity) experience greater within-session social satiety but only for same-sex and not opposite-sex stimuli.

Table S7. Correlation matrix of behavioural economics outcomes with social rewards and video-derived open-door outcomes from Phases 2 and 3.

Social operant outcome	Phase 2 FR	Mean adjusted social rewards		Total time spent nose in open door		Proportion of open door time with nose in door	
		Pearson's *r*	*p*	Pearson's *r*	*p*	Pearson's *r*	*p*
Demand at null cost (Q0)	*1*	0.448	0.025	0.559	0.001	-0.035	0.851
	2	0.313	0.128	0.276	0.126	0.127	0.497
	4	0.458	0.008	0.318	0.081	0.224	0.226
	6	0.571	0.001	0.588	< 0.001	-0.442	0.011
	9	0.667	< 0.001	0.686	< 0.001	-0.360	0.043
	12	0.624	< 0.001	0.741	< 0.001	-0.320	0.074
Demand elasticity (α)	*1*	-0.327	0.111	-0.392	0.027	-0.112	0.550
	2	-0.225	0.215	-0.218	0.238	-0.312	0.087
	4	-0.389	0.028	-0.273	0.130	-0.054	0.769
	6	-0.398	0.024	-0.356	0.045	0.170	0.352
	9	-0.429	0.014	-0.380	0.032	0.159	0.383
	12	-0.416	0.018	-0.394	0.025	0.429	0.014

Social operant outcome	Phase 3	Mean adjusted social rewards		Total time spent nose in open door		Proportion of open door time with nose in door	
		Pearson's *r*	*p*	Pearson's *r*	*p*	Pearson's *r*	*p*
Demand at null cost (Q0)	*Same*	0.314	0.097	0.272	0.154	-0.102	0.597
	Opposite	0.056	0.772	0.297	0.118	0.068	0.727
Demand elasticity (α)	*Same*	-0.850	< 0.001	-0.380	0.042	0.475	0.009
	Opposite	-0.840	< 0.001	-0.467	0.007	0.136	0.459

6. A guide to common experimental parameters in social operant conditioning

To summarise the practical implications of this study for research utilising the social operant conditioning model to assess social motivation (alone or as an alternative to motivation for drug or food reward in a choice paradigm), we have included the following list of considerations:

1. *Biological sex:*
    a. When designing social operant conditioning experiments, sex differences in social motivation can emerge; mixed-sex cohorts of sufficient sample size to detect sex differences should be considered during experimental design.
    b. While tracking oestrus phase in female experimental and stimulus rats is optimal, it is not necessary for clear interpretation of social operant data or to justify inclusion of female subjects in social operant conditioning.
2. *Housing:*
    a. Individual housing of experimental subjects is not necessary for acquisition and expression of social operant responding in this model.
    b. However, if greater social motivation is required to establish reliable responding, individual housing of subjects should enhance drive for social interaction.
        i. As a caveat, isolated housing may increase social motivation in female more than male rats, and the increase for male rats may be negligible.
3. *Stimulus sex:*
    a. Rats can reliably express operant responding for social interaction with both same- and opposite-sex stimulus partners.
    b. However, if greater motivation is required to establish reliable operant responding, using opposite-sex stimulus partners should elevate motivation for social interaction.

            i. As a caveat, motivation for opposite-sex stimulus partners may involve indistinct contributions of social and sexual motivation, although it more likely reflects a primarily social drive.

4. *Time-of-day and circadian factors:*
    a. While it is ethologically preferrable to conduct social operant conditioning during the early-dark phase when rats are typically most active, this is not critical to rats acquiring and expressing social operant responding.
    b. Regardless of the ToD chosen for social testing, testing should be maintained consistently within this selected circadian phase.
5. *Video recording and analyses:*
    a. Whenever possible, video recording and analyses should be implemented alongside classical operant outcomes during social operant conditioning to fully characterise the impact of interventions on social operant behaviour, and to delineate between motivation for social interaction, and social interaction itself.
    b. Video recording should primarily focus on capturing interactions between the experimental rat and the social door.
        i. Outcomes of interest may include total time spent with nose in the open/closed door, proportion of open-door/closed-door time with nose in door, bouts of interaction etc.
    c. Additional aspects of interest derived from video recording may pertain to general movement (e.g., total duration, speed, and immobility) and interaction of subjects with cues and levers (i.e., distinguishing sign- vs goal-tracking subjects).
6. *Behavioural economics:*
    a. If one's primary aim is to assess the effect of an intervention on social demand at null cost, a between-session paradigm may be more appropriate.

b. If one’s primary aim is to assess the effect of an intervention on social demand elasticity, a within-session paradigm may be more appropriate.

c. To successfully apply behavioural economics to the social operant conditioning model, selection of the top 50% of socially motivated subjects (as determined using data from acquisition) may be necessary.

   i. For predicting future social operant performance of subjects for between-session behavioural economics from acquisition data, outcomes of higher social rewards and locomotion from acquisition sessions 3-4 onwards best predict Q_0 and α (Table S3).

   ii. For predicting future social operant performance of subjects for within-session behavioural economics from acquisition data, outcomes of higher social rewards, locomotion, total time spent with nose in open door, and active-inactive difference from acquisition Sessions 2-3 onwards best predict Q_0 and α (See section 4.1 and Table S4 for further details).

7. *Stimulus cycling:*

   a. This study did not directly compare whether stimulus cycling elevates social motivation in experimental rats compared to using the same stimulus consistently, thus it cannot inform whether this practice is effective.

   b. Based on preliminary analyses conducted during Phase 1 (see Figure S7), it is possible that individual differences in stimulus rats’ behaviour during social operant sessions does not substantially impact experimental rats’ social motivation for interaction with stimulus rats.

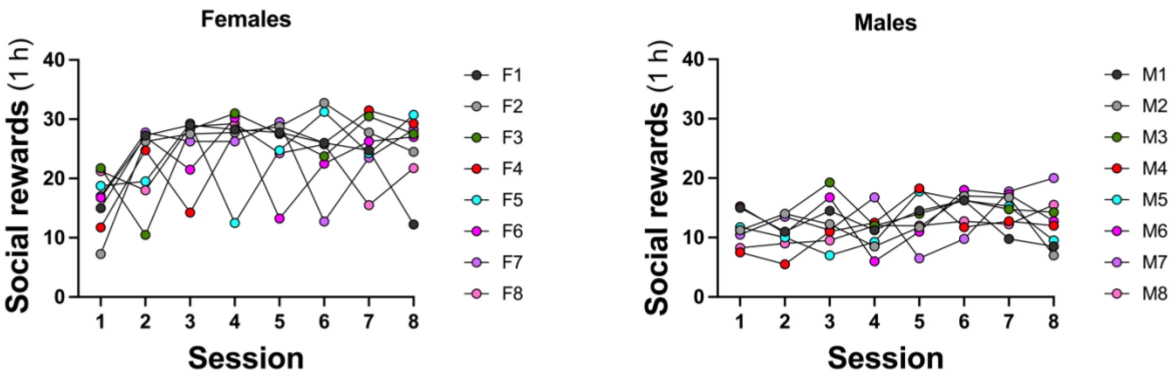

Figure S7. Influence of stimulus rat on social rewards earned during Phase 1 (Acquisition). Averaged across Sessions 1-8, no effect of stimulus animal on social rewards obtained was identified for females [$F(5.189, 36.32) = 0.1580$, $p = 0.9785$] nor males [$F(3.209, 22.46) = 0.7672$, $p = 0.5322$] (one-way repeated-measures ANOVA). Further, no individual pairwise comparisons between stimulus rats were significant (all $p > 0.05$) indicating that no one stimulus animal acted as an inferior or superior social stimulus in the context of social reward data. Data represent individual subject data of social rewards obtained; each stimulus rat is represented by colour and connected by solid lines. As Phase 1 only included same-sex conspecific stimulus interactions, data are separated into female-female and male-male social interaction sessions.

7. References

1. Butler-Struben HM, Kentner AC, Trainor BC. What's wrong with my experiment?: The impact of hidden variables on neuropsychopharmacology research. Neuropsychopharmacology. 2022;47(7):1285-91.
2. Francis NA, Kanold PO. Automated operant conditioning in the mouse home cage. Frontiers in neural circuits. 2017;11:10.
3. Mistlberger RE, Skene DJ. Social influences on mammalian circadian rhythms: animal and human studies. Biological Reviews. 2004;79(3):533-56.
4. Elmore SK, Betrus PA, Burr R. Light, social zeitgebers, and the sleep–wake cycle in the entrainment of human circadian rhythms. Research in nursing & health. 1994;17(6):471-8.
5. Ehlers CL, Frank E, Kupfer DJ. Social zeitgebers and biological rhythms: a unified approach to understanding the etiology of depression. Archives of general psychiatry. 1988;45(10):948-52.
6. Grandin LD, Alloy LB, Abramson LY. The social zeitgeber theory, circadian rhythms, and mood disorders: review and evaluation. Clinical psychology review. 2006;26(6):679-94.
